# Metastatic Triple Negative Breast Cancer: Navigating a Rapidly Evolving Therapeutic Landscape

**DOI:** 10.32604/or.2026.082829

**Published:** 2026-09-14

**Authors:** Iseult M. Browne, Monica Esteban Garcia, Alicia F. C. Okines

**Affiliations:** 1 The Breast Unit, The Royal Marsden Hospital NHS Foundation Trust, London, UK; 2 The Breast Cancer Now Toby Robins Research Centre, The Institute of Cancer Research, London, UK

**Keywords:** Triple negative breast cancer, antibody-drug conjugates, immune checkpoint inhibition, PARP inhibitors, biomarkers, *BRCA* mutation, treatment sequencing, sacituzumab govitecan, pembrolizumab

## Abstract

Triple negative breast cancer (TNBC) is defined by the absence of oestrogen receptor, progesterone receptor, and HER2 expression, and carries a disproportionate burden of breast cancer-related mortality due to its aggressive biology and historically limited therapeutic options. The treatment landscape of metastatic TNBC has undergone a fundamental transformation over the past decade, driven by immune checkpoint inhibitors, antibody-drug conjugates (ADCs), and the identification of actionable genomic alterations. This review provides a comprehensive, clinically oriented appraisal of the current and emerging therapeutic landscape of metastatic TNBC, encompassing its molecular underpinnings and tumour microenvironment biology. We critically evaluate the evolving roles of immune checkpoint inhibition, ADCs, PARP inhibitors, and novel targeted approaches, discuss mechanisms of resistance and biomarker limitations, and propose a framework for rational, biomarker-guided treatment selection and sequencing to support evidence-based clinical decision-making in this rapidly evolving field.

## 1 Introduction

### 1.1 Epidemiology and Clinical Significance

Breast cancer remains the most frequently diagnosed malignancy worldwide, with an estimated 2.3 million new cases and 685,000 deaths reported globally in 2020 [1]. Triple negative breast cancer (TNBC), which is characterised by the absence of ER, PR, and *HER2* amplification or overexpression, accounts for approximately 10–15% of all breast cancers, translating to approximately 300,000 cases annually [2]. Despite constituting a minority of diagnoses, TNBC is responsible for a disproportionate share of breast cancer-specific mortality, a disparity attributable to its aggressive clinical behaviour, high rates of visceral and central nervous system (CNS) metastasis, and historically limited therapeutic options [3].

This subtype is enriched amongst younger women, women of African ancestry, and those with germline *BRCA* gene mutations [4,5]. The peak incidence is between 40 and 50 years of age; approximately a decade younger than the median age at diagnosis of the most common, hormone receptor-positive (HR-positive) subtype [5]. *BRCA1/2*-associated TNBC constitutes approximately 10–20% of all TNBC cases, underscoring the importance of germline testing in this population [6]. Population-based analyses have demonstrated that Black women have a two-fold higher incidence of TNBC compared with White women, an observation partly attributable to germline susceptibility variants; socioeconomic factors, meanwhile, influence access to timely treatment [5,7].

In the metastatic setting, TNBC carries a substantially worse prognosis than other breast cancer subtypes, in part due to the successful development of HER2-targeted agents. Prior to the introduction of immunotherapy for PD-L1-positive TNBC, the median progression-free survival (PFS) with first-line chemotherapy ranged from 5 to 8 months, and median overall survival (OS) was approximately 12 to 18 months [8]. The five-year survival rate for metastatic TNBC is estimated at less than 12%, in stark contrast to HER2-positive or HR-positive disease, where longer-term survival is increasingly achievable [9]. TNBC is associated with a high risk of visceral metastases, particularly to the lungs and liver, and carries a notable risk of CNS involvement, with brain metastases reported in up to 50% of patients with metastatic TNBC [10,11].

### 1.2 Challenges in Management

The management of metastatic TNBC is complicated by several interrelated challenges. Firstly, the defining absence of ER, PR, and HER2 renders endocrine therapy and HER2-directed agents ineffective, historically confining systemic treatment options to cytotoxic chemotherapy [12]. Secondly, TNBC exhibits profound molecular heterogeneity which we will discuss in more detail later in this review. Even within the triple-negative designation, multiple distinct molecular subtypes exist with divergent gene expression profiles, proliferative indices, immune infiltrates, and oncogenic dependencies [13]. This heterogeneity complicates biomarker-driven patient selection and undermines the applicability of a one-size-fits-all chemotherapeutic approach. Thirdly, the disease course is characteristically aggressive with rapid progression on therapy, early development of acquired resistance to cytotoxic agents, and accumulation of treatment-related toxicities despite a relatively young patient population [3].

Brain metastases represent a major cause of morbidity and mortality in metastatic TNBC, with the median interval from diagnosis of metastatic disease to CNS involvement in the order of approximately 10–18 months in retrospective analyses, reflecting the often early onset of brain relapse in this subtype and often limited survival beyond brain metastases diagnosis [11]. The historical management of brain metastases in TNBC relied predominantly on local therapy, largely comprising whole-brain radiotherapy (WBRT) for the often extensive CNS involvement. Systemic therapy options were severely limited by poor blood brain barrier (BBB) penetration of conventional chemotherapeutic agents; for example doxorubicin and taxanes achieve cerebrospinal fluid (CSF) concentrations well below therapeutic thresholds [14]. Historically, patients with active brain metastases were excluded from clinical trials, creating a profound evidence gap. The emergence of CNS-penetrant agents, including newer-generation antibody-drug conjugates (ADCs) and small molecule inhibitors, has begun to address this need, as we will discuss in subsequent sections.

### 1.3 Rationale for This Review

The past five years have marked a period of significant evolution in the management of TNBC, with multiple landmark clinical trials reshaping available therapeutic options. The KEYNOTE-355 and IMpassion130 trials established immunotherapy, specifically PD-L1-directed immune checkpoint inhibition (ICI) in combination with chemotherapy, as a new first-line standard of care for PD-L1-positive disease [15,16]. The ASCENT trial demonstrated the superiority of the TROP-2-directed ADC sacituzumab govitecan (SG) over single-agent chemotherapy in previously treated disease [17]. Concurrently, the emergence of HER2-low as a therapeutically relevant biomarker, validated by the DESTINY-Breast04 trial, has identified a new targetable subset within TNBC [18].

More recently, ADCs are being evaluated earlier in the disease course, including in the first-line setting. Following the survival advantage demonstrated with SG in previously treated metastatic TNBC, subsequent studies have investigated its integration into frontline therapy. ASCENT-03 evaluated SG versus physician’s choice chemotherapy in the first-line setting for PD-L1-negative, or ICI ineligible, metastatic TNBC, while ASCENT-04 assessed SG in combination with pembrolizumab for PD-L1-positive disease [19,20]. Reported data from these trials demonstrate clinically meaningful improvements in PFS compared with chemotherapy-based standards, supporting the potential repositioning of ADCs into earlier lines of therapy. This is particularly important given only approximately 50% of patients with metastatic TNBC will receive a second line of palliative therapy [21]. In parallel, TROPION-Breast02 evaluated the TROP-2-targeting ADC datopotamab deruxtecan (Dato-DXd) versus chemotherapy in first line ICI-ineligible patients with metastatic TNBC, reporting both improved PFS and OS in patients unsuitable for ICIs, with potential implications for future sequencing strategies [22].

Collectively, these findings signal a paradigm shift in which ADCs are no longer confined to the post-chemotherapy setting but are emerging as candidates for frontline therapy. This transition necessitates careful consideration of optimal integration with immune checkpoint blockade, mechanisms of primary and acquired resistance, biomarker refinement, and the management of ADC-specific toxicities as these agents are incorporated earlier in the treatment algorithm.

Against this backdrop, the pipeline of emerging agents, encompassing novel ADCs, next-generation PARP inhibitors, AKT pathway inhibitors, androgen receptor antagonists, and early-phase cell-based therapies, continues to expand rapidly.

This review systematically appraises the evolving treatment landscape of metastatic TNBC, from the molecular underpinnings of the disease to the clinical evidence supporting current and emerging therapeutic strategies. This review aims to provide a clinically actionable framework to support oncologists in navigating this increasingly complex and rapidly evolving therapeutic landscape.

Throughout this review, trial selection prioritises phase III randomised evidence in the metastatic TNBC setting, supplemented by pivotal phase I/II data where phase III evidence is unavailable or where early-phase findings are considered practice-informing. Trials conducted in broader breast cancer populations are included where directly relevant to biomarker-defined subgroups that overlap with TNBC, such as HER2-low and germline *BRCA*-mutated disease.

## 2 Pathophysiology and Molecular Characteristics

### 2.1 Molecular Subtypes of TNBC

Gene expression profiling studies have delineated several distinct molecular subtypes within the broad TNBC category, most comprehensively characterised by the Lehmann classification, which identified six TNBC subtypes: basal-like 1 (BL1), basal-like 2 (BL2), mesenchymal (M), mesenchymal stem-like (MSL), immunomodulatory (IM), and luminal androgen receptor (LAR) [23]. Subsequently, a refined four-subtype model consolidated overlapping transcriptomic profiles into BL1, BL2, M, and LAR subtypes, each with distinct oncogenic dependencies and potential therapeutic vulnerabilities [13].

BL1 tumours, the most common subtype, are characterised by high expression of cell cycle genes (CDK1/2), DNA damage response genes (*BRCA1*, *TP53BP1*), and elevated proliferative indices. These tumours demonstrate the highest pathological complete response (pCR) rates to platinum-based neoadjuvant chemotherapy (NACT) and are the most immunologically active [24]. BL2 tumours express growth factor signalling components (EGFR, NGF, MET) and IGF1R, conferring relative resistance to conventional chemotherapy. The mesenchymal subtype (M) expresses genes involved in cell motility and the epithelial-mesenchymal transition (EMT), and exhibits activation of the PI3K/mTOR and Wnt/β-catenin pathways, providing a rationale for targeted inhibition of these pathways [13]. The LAR subtype overexpresses androgen receptor (AR) and luminal cytokeratins, and molecular profiling reveals *PIK3CA* mutations in approximately 50% of cases, suggesting dual AR and PI3K pathway targeting as a rational therapeutic strategy [25].

Beyond the Lehmann classification, additional molecular features further stratify TNBC. Claudin-low tumours are enriched for cancer stem cell characteristics, EMT gene signatures, and immune cell infiltrates, and are associated with relatively chemotherapy-resistant biology [26]. An interferon-rich subgroup, characterised by high expression of innate immune pathway genes and type I interferon signalling, has been associated with the highest degree of immune infiltration and identified as the subtype most likely to respond to ICI [27]. A thorough understanding of these molecular subtypes is essential for guiding rational, biomarker-driven therapeutic selection.

### 2.2 Somatic Mutations and Key Biomarkers

*TP53* mutations are the most prevalent somatic genetic alterations in TNBC, occurring in approximately 65–80% of cases, compared with 20–30% in HR+ breast cancer [28]. Loss of *TP53* function promotes genomic instability, accelerates cell cycle progression, and contributes to the aggressive clinical phenotype. *PIK3CA* mutations are identified in approximately 8–10% of TNBC, and are particularly enriched in the LAR subtype, where they may be detected in up to 40-50% of cases, activating the PI3K/AKT/mTOR pathway and providing a potential pharmacological target [25]. *PTEN* loss, occurring through deletion or somatic mutation in up to 25–35% of TNBC cases, similarly activates PI3K/AKT signalling [29] and may independently predict sensitivity to AKT inhibitors, although only modest activity was reported with capivasertib in patients with ER-negative disease and *AKT* or *PTEN* mutations in the UK plasmaMATCH trial [30].

Germline *BRCA1/2* mutations, present in 10–20% of TNBC, disrupt homologous recombination (HR) DNA repair, rendering tumours acutely sensitive to PARP inhibitors and platinum-based chemotherapy [6]. Somatic *BRCA1/2* mutations and alterations in other HR pathway genes, collectively referred to as homologous recombination repair (HRR) alterations, (*RAD51*, *PALB2*, *ATM*) contribute to the broader ‘BRCAness’ phenotype, characterised by homologous recombination deficiency (HRD), which may extend sensitivity to PARP inhibitors beyond patients with germline *BRCA* mutations [31]. Benefit was limited to patients with germline or somatic *BRCA1/2* or *PALB2* mutations in the TBCRC-048 trial of olaparib, with no responses reported in patients with *ATM* or *CHEK2* mutations [32]. *FGFR2* amplification, which occurs in approximately 4% of TNBC, activates receptor tyrosine kinase (RTK) signalling and represents an emerging therapeutic target given preclinical sensitivity to FGFR inhibitors [33]. However clinical evaluation has been disappointing [34]. In contrast, *HER2* (*ERBB2*) mutations, distinct from HER2 amplification, are identified in 2–5% of TNBC and have demonstrated actionability with useful response rates reported for neratinib, tucatinib with trastuzumab and trastuzumab deruxtecan (T-DXd) [35,36,37] with the tucatinib combination recently included in NCCN guidelines.

PD-L1 expression, assessed by immunohistochemistry (IHC) using the SP142 antibody (immune cell [IC] score ≥1%) or the 22C3 antibody (Combined Positive Score [CPS] ≥10), is the principal predictive biomarker for ICI benefit in the first-line metastatic setting [15,16].

AR expression, defined by IHC positivity ≥10% in most studies, characterises approximately 20–30% of TNBC and identifies the LAR subtype as a candidate for androgen receptor pathway inhibition [23]. Attempts to re-purpose anti-androgens including enzalutamide [38] and abiraterone [39] in AR-positive TNBC have reported modest clinical benefit, possibly affected by the use of IHC rather than RNA expression for patient selection [40].

TROP-2 is highly expressed in the majority of TNBC (>85%), however TROP-2 expression is not currently used as a predictive biomarker for patient selection in clinical practice [41].

### 2.3 The Tumour Microenvironment and Therapeutic Implications

The tumour microenvironment (TME) of TNBC is uniquely dynamic and exerts a profound influence on treatment responsiveness. TNBC harbours the highest density of tumour-infiltrating lymphocytes (TILs) of all breast cancer subtypes, with high stromal TIL levels (>50%) identified in approximately 20–25% of cases [42]. TIL density is both prognostic for survival and predictive of response to chemotherapy and immunotherapy [43]. The predictive value of TILs for ICI benefit is particularly evident in the context of PD-L1 co-expression, and tumours with high TIL density and PD-L1 positivity represent the immunological phenotype most likely to benefit from checkpoint blockade [44].

Tumour-associated macrophages (TAMs) constitute a major TME component in TNBC and exist along a spectrum from pro-inflammatory M1 to immunosuppressive M2 phenotypes. M2-polarised TAMs, characterised by expression of CD163, CD206, and secretion of IL-10 and TGF-β, promote tumour invasion, angiogenesis, and immune evasion, and are associated with poorer clinical outcomes [45]. The ratio of M1 to M2 TAMs within the TME has been proposed as a prognostic and predictive biomarker with high M2 TAM infiltration correlating with resistance to both chemotherapy and immunotherapy [46]. Strategies modulating TAM polarisation, including CSF1R inhibitors, CD47 blockade, and TGF-β pathway inhibition, are under active investigation as combination partners for established therapies [47,48,49].

Cancer-associated fibroblasts (CAFs) contribute to the desmoplastic stroma that characterises a subset of TNBC, creating a physical barrier to drug penetration and secreting immunosuppressive cytokines including CXCL12, which excludes cytotoxic T lymphocytes from the tumour core [50]. Neutrophil infiltration, particularly tumour-associated neutrophils (TANs) with an N2 phenotype, further contributes to immune suppression and has been associated with worse outcomes in metastatic TNBC [51]. The interplay between these cellular components underscores the complexity of TME-directed therapeutic strategies and the rationale for combinatorial approaches targeting both the tumour cell and its immunological niche.

## 3 Historical Standard of Care

### 3.1 Chemotherapy as the Therapeutic Backbone

For the majority of the past three decades, cytotoxic chemotherapy represented the sole systemic therapeutic option for metastatic TNBC. The backbone agents including anthracyclines (doxorubicin, epirubicin), taxanes (paclitaxel, docetaxel, nab-paclitaxel), and platinum compounds (carboplatin, cisplatin), were extrapolated from broader metastatic breast cancer experience and applied sequentially in the metastatic setting [52]. The clinical benefit of each agent in TNBC was established through retrospective subset analyses and relatively modest prospective data (Table 1).

**Table 1:** Standard Chemotherapy Regimens for Metastatic TNBC.

Regimen	Setting	ORR	mPFS	mOS	Key Trial/Clinical Notes
**Taxanes (paclitaxel, docetaxel, nab-paclitaxel)**	1L/2L	25–35%*	5–7 m*	~12-15 m*	Common chemoimmunotherapy backbone (KEYNOTE-355: nab-paclitaxel, paclitaxel; IMpassion130: nab-paclitaxel). Peripheral neuropathy key cumulative toxicity
**Gemcitabine + carboplatin**	1L/2L	32-44%^a^	4.1–6.0 m^a^	~11-13 m^a^	Common chemoimmunotherapy backbone (KEYNOTE-355, ASCENT-03, ASCENT-04 control arms). Carboplatin has enhanced activity in g*BRCA*-mutated disease
**Anthracyclines (doxorubicin, epirubicin; AC/EC/FEC)**	1L (if anthracycline-naïve)	20–30%*	6–8 m*	~16-18 m*	Rarely used in contemporary metastatic practice given widespread prior (neo)adjuvant exposure. Cumulative cardiotoxicity limits lifetime dosing. Relevant for patients with *de novo* metastatic disease who have not received prior anthracyclines
**Capecitabine**	2L+	15–29%*	3–5 m*	~13 m*	Modest response rates in heavily pretreated disease. Commonly used as TPC comparator (OlympiAD, EMBRACA, KEYNOTE-119). Useful in patients with residual neuropathy limiting taxane use
**Eribulin**	2L+	11–30%^*^	2.7–4.1 m^b^	12.9 m^b^	Common TPC comparator. Relatively lower neuropathy compared with taxanes
**Vinorelbine**	2L+	4-10%*	1.4–3 m*	~6-10 m*	Modest activity in pretreated disease; used as TPC comparator (OlympiAD, EMBRACA). Oral formulation available. Less neuropathy than taxanes.
**Gemcitabine (single agent)**	2L+	12–17%*	3–4 m*	~8-11 m*	Modest monotherapy activity; preferred in patients with residual neuropathy or inability to tolerate taxanes or platinum combinations. Commonly used as TPC option

*Range derived from retrospective subgroup analyses and single-arm studies in TNBC-enriched or mixed populations; formal phase III data in TNBC specifically are limited for these agents. Figures should be interpreted with caution given heterogeneous trial populations and prior treatment exposures. ^a^G/C figures derived from the G/C control arms of tnAcity (Yardley, *Ann Oncol*. 2018) (phase II, first-line mTNBC; ORR 44%, PFS 6.0 m, OS 12.6 m) and O'Shaughnessy et al. phase III trial of G/C ± iniparib in mTNBC (O'Shaughnessy, *J Clin Oncol*. 2014) (G/C alone arm: PFS 4.1 m, OS 11.1 m). ^b^TNBC-specific data from pooled analysis of EMBRACE and Study 301 (Twelves, *Breast Cancer Res Treat*. 2014) eribulin OS 12.9 m vs. control 8.2 m (HR 0.74; *p* = 0.006); PFS 2.8 m. TNBC-specific ORR not separately reported. G/C, gemcitabine/carboplatin; gBRCA, germline BRCA; m, months; mOS, median overall survival; mPFS, median progression-free survival; ORR, objective response rate; TPC, treatment of physician's choice; TNBC, triple-negative breast cancer; 1L, first line; 2L, second line.

Anthracycline agents historically demonstrated modest single-agent activity in metastatic breast cancer overall, with response rates in the range of 30–40%, with subgroup analyses in metastatic TNBC suggesting ORRs in the first-line setting generally lie within the 20–30% range [53]. Taxanes similarly demonstrated ORRs of 25–35%, with weekly paclitaxel offering superior tolerability compared with three-weekly schedules [54]. Combination with the VEGF-A monoclonal antibody, bevacizumab, improved response rate and PFS [55] but not overall survival [56]. Nab-paclitaxel (albumin-bound paclitaxel) demonstrated particular relevance in TNBC in the neoadjuvant setting, and the GeparSepto trial showed superior pathological complete response (pCR) rates with nab-paclitaxel compared with paclitaxel, particularly in TNBC [57]. In combination, anthracycline-taxane doublets formed the basis of standard NACT regimens.

Platinum agents have particular relevance in TNBC given the high prevalence of *BRCA1/2* mutations and underlying HRD. In the neoadjuvant setting, the phase III BrighTNess trial demonstrated that adding carboplatin to paclitaxel followed by doxorubicin/cyclophosphamide significantly improved pCR rates (58% vs. 31%, *p* < 0.0001) [58]. Similarly, the GeparSixto trial reported significantly improved pCR rates with the addition of carboplatin to an anthracycline-taxane backbone in TNBC [59], leading to standard incorporation of carboplatin into neo-adjuvant regimens. In the metastatic setting, retrospective analyses suggest platinum compounds may be particularly effective in tumours with *BRCA1/2* mutations. The TNT trial, a randomised phase III study, showed that carboplatin conferred higher ORRs than docetaxel specifically in patients with germline *BRCA1/2*-mutated metastatic breast cancer (68% vs. 33%, *p* = 0.03), informing current treatment algorithms that favour platinum in this subgroup [60]. Later-line systemic options such as capecitabine and eribulin are widely used in metastatic TNBC. The phase III EMBRACE trial established an OS benefit for eribulin monotherapy compared with treatment of physician’s choice in heavily pretreated breast cancer, including a TNBC subgroup (13.1 vs. 10.6 months; HR 0.81, 95% CI 0.67–0.96; *p* = 0.041) [61].

### 3.2 Limitations of Conventional Chemotherapy

Despite representing the standard of care for decades, conventional chemotherapy in metastatic TNBC is characterised by several critical limitations. The durability of response is modest, median PFS with first-line chemotherapy regimens rarely exceeds 6–8 months, and acquired resistance develops almost universally [8]. The mechanisms of chemotherapy resistance in TNBC are multifaceted, encompassing overexpression of drug efflux pumps (P-glycoprotein/ABCB1), upregulation of anti-apoptotic proteins (BCL-2, BCL-XL), enrichment of cancer stem cell populations, and alterations in the TME that promote immune evasion and drug sequestration [62]. These resistance mechanisms accumulate with successive lines of therapy, explaining the progressively diminishing response rates observed across treatment lines.

Cumulative toxicity represents an additional constraint. Anthracycline-associated cardiotoxicity limits total lifetime dosing, and patients with prior anthracycline exposure in the (neo)adjuvant setting often cannot receive further anthracycline therapy in the metastatic setting [63]. Taxane-induced peripheral neuropathy causes significant and often irreversible functional impairment, particularly limiting in a young patient population [64]. Platinum-associated nephrotoxicity, myelosuppression, and neurotoxicity further limit dosing and duration of therapy. The cumulative impact of these toxicities on quality of life (QoL) is substantial and, until recently, had to be weighed against modest and time-limited clinical benefits.

## 4 Immunotherapy in Triple-Negative Breast Cancer

### 4.1 Pivotal Trials in First-Line Setting

The incorporation of ICIs into the therapeutic landscape of metastatic TNBC represents a major advance, although meaningful benefit remains largely restricted to biomarker-selected populations and specific disease settings. Among available biomarkers, PD-L1 expression remains the only clinically validated predictive marker in the first-line metastatic setting. Assay selection and scoring methodology differ materially across pivotal trials, complicating cross-study comparisons. IMpassion130 defined PD-L1 positivity using the SP142 assay with immune-cell (IC) staining ≥1%, whereas KEYNOTE-355 employed the 22C3 assay and combined positive score (CPS), with the most pronounced benefit observed in tumours with CPS ≥10 [15,16,44,65]. Despite differences in methodology, both trials consistently demonstrated that clinically meaningful benefit from chemo-immunotherapy is largely confined to PD-L1-positive disease in the first line setting.

The phase III IMpassion130 trial (NCT02425891) provided proof-of-concept for ICIs in metastatic TNBC. In patients with PD-L1-positive tumours (IC ≥1%), the addition of the anti-PD-L1 antibody, atezolizumab, to nab-paclitaxel significantly improved PFS, with a median of 7.5 months versus 5.0 months for chemotherapy alone (HR 0.62; 95% CI 0.49–0.78; *p* < 0.001) [16]. A clinically meaningful OS improvement was also observed in this subgroup, with median OS of 25.4 versus 17.9 months (HR 0.67; 95% CI 0.53–0.86), although this did not reach formal statistical significance given the hierarchical testing design [65]. These findings provided the conceptual and regulatory basis for first-line chemo-immunotherapy in PD-L1 selected disease. In contrast, in IMpassion131 (NCT03125902), atezolizumab combined with paclitaxel failed to improve PFS in the PD-L1-positive population (HR 0.82; 95% CI 0.60–1.12; *p* = 0.20) or OS [66].

The discordant results between IMpassion130 and IMpassion131 have not been definitively explained. Routine corticosteroid premedication required with paclitaxel has been proposed as a potential immunosuppressive confound, though this hypothesis is not supported by subgroup analysis of KEYNOTE-355 (NCT02819518), in which no reduction in efficacy was observed in patients who received paclitaxel with pembrolizumab [67]. Additional proposed explanations include differences in baseline patient characteristics between the two trials and intrinsic pharmacological differences between nab-paclitaxel and solvent-based paclitaxel in the tumour immune microenvironment, raising the possibility that the atezolizumab-paclitaxel combination has inferior immunostimulatory activity. KEYNOTE-355 ultimately established pembrolizumab plus chemotherapy as the preferred first-line regimen for PD-L1-positive (CPS ≥10) metastatic TNBC, reporting a significant improvement in median PFS in patients with PD-L1 CPS ≥10, (9.7 months versus 5.6 months with chemotherapy alone, HR 0.65; 95% CI 0.49–0.86; *p* = 0.0012), and OS, (median 23.0 versus 16.1 months, HR 0.73; 95% CI 0.55–0.95; *p* = 0.0185) [67]. Benefit was attenuated at lower CPS thresholds, reinforcing the need for rigorous PD-L1 assessment prior to treatment selection. Notably, atezolizumab plus nab-paclitaxel received accelerated approval from the FDA based on IMpassion130 but was later withdrawn after IMpassion131 failed to confirm an OS benefit, whereas the European Medicines Agency continues to maintain approval in this setting for patients with PD-L1-positive tumours (IC ≥1%). Key clinical trials of ICIs in metastatic TNBC can be found in Table 2. Combination trials of ICIs with ADCs can be found in Table 3.

**Table 2:** Key Clinical Trials of Immune Checkpoint Inhibitors in Metastatic TNBC.

Trial	Treatment Arms	PDL1	Line	ORR (PD-L1+)	mPFS (PD-L1+)	mOS (PD-L1+)	Approval Status
**IMpassion130** (NCT02425891)	Atezo + nab-P vs. Pbo + nab-P	IC ≥ 1% (SP142)	1L	59% v 43%	7.5 v 5.0 m (HR 0.62)	25.4 vs. 17.9 m (HR0.67ª)	FDA accelerated approval 2019 IC≥1% (withdrawn 2021); EMA approval maintained
**IMpassion131** (NCT03125902)	Atezo + paclitaxel vs. Pbo + paclitaxel	IC ≥ 1% (SP142)	1L	63% vs. 55%	6.0 vs. 5.7 m (HR 0.82)	22.1 vs. 28.3 m (HR 1.11)	Negative trial
**KEYNOTE-355** (NCT02819518)	Pembro + CT vs. Pbo + CT (paclitaxel, nab-paclitaxel, gemcitabine/carboplatin)	CPS ≥ 10 (22C3)	1L	53% vs. 40%	9.7 vs. 5.6 m (HR 0.65)	23.0 vs. 16.1 m (HR 0.73)	FDA/EMA approved (CPS ≥10)
**KEYNOTE-119** (NCT02555657)	Pembro mono vs. CT (capecitabine, eribulin, vinorelbine, gemcitabine)	CPS ≥ 1/≥ 10 (22C3)	2L/3L	9.6% (ITT) ^b^	Not formally tested ^b^	CPS ≥ 10: 12.7 vs. 11.6 m (HR 0.78)^b^	Negative trial
**KEYNOTE-086 (Cohort B)** (NCT02447003)	Pembro monotherapy (single-arm)	CPS ≥ 1 (22C3)	1L	23%	2.1 m	16.1 m	No regulatory approval in 1L
**TORCHLIGHT** (**NCT03777579**)	Toripalimab + nab-P vs. Pbo + nab-P	CPS ≥ 1 (JS311)	1L	66% vs. 68%	8.4 vs. 5.6 m (HR 0.65)	32.8 vs. 19.5 m (HR 0.62)	Approved in China only

^a^In *IMpassion130*, OS in the PD-L1 IC ≥1% subgroup was evaluated as part of a hierarchical testing strategy. Although the hazard ratio favored atezolizumab, the endpoint did not meet the prespecified threshold for statistical significance due to multiplicity control; therefore, the result should be considered exploratory. ^b^*KEYNOTE-119* did not meet its primary endpoint of OS in the intention-to-treat (ITT) population (HR 0.97; *p* = 0.74). Analyses in PD-L1–defined subgroups (CPS ≥1 and CPS ≥10), including ORR and PFS, were exploratory and not adjusted for multiplicity due to failure of the primary endpoint. The OS result in the CPS ≥10 subgroup represents a post hoc exploratory analysis. Atezolizumab (*Atezo*); chemotherapy (*CT*); combined positive score (*CPS*); European Medicines Agency (*EMA*); US Food and Drug Administration (*FDA*); immune cell score (*IC*); intention-to-treat (*ITT*); months (*m*); median overall survival (*mOS*); median progression-free survival (*mPFS*); nab-paclitaxel (*nab-P*); objective response rate (*ORR*); placebo (*Pbo*); pembrolizumab (*Pembro*); programmed death-ligand 1 (*PD-L1*); first-line (*1L*); second-line (*2L*); third-line (*3L*).

### 4.2 PD-L1 Inhibition in Later Lines

The role of single-agent ICI in previously treated metastatic TNBC is limited. The phase III KEYNOTE-119 study (NCT02555657) evaluated pembrolizumab monotherapy versus treatment of physicians choice (TPC) single-agent chemotherapy in patients with two or more prior lines of therapy for metastatic disease [68]. The trial did not meet its primary endpoint: OS was not improved in the overall population (HR 0.97; 95% CI 0.82–1.15; *p* = 0.74), and although a trend toward benefit was observed in the CPS ≥10 subgroup (HR 0.78; 95% CI 0.57–1.06; *p* = 0.057), this did not achieve statistical significance. Notably, the ORR with pembrolizumab monotherapy was only 9.6% in the overall population. This figure is consistent with earlier phase II data from KEYNOTE-086 cohort A, in which single-agent pembrolizumab yielded an ORR of 5.3% (95% CI 2.7–9.9%) in previously treated TNBC, with no meaningful enrichment by PD-L1 status [69]. Together, these data indicate that the predictive utility of PD-L1 expression is substantially attenuated in the pretreated setting, and that single-agent PD-L1 blockade beyond the first line produces clinically meaningful responses in only a small minority of patients.

Response patterns across trials therefore reveal a consistent gradient of benefit according to line of therapy and the greatest efficacy is observed when ICI is introduced in the first-line metastatic setting in PD-L1-positive disease, with activity diminishing progressively in later lines. This potentially reflects depletion of tumour-reactive T-cell clones, progressive immunological attrition, and remodelling of the tumour microenvironment toward an immunosuppressed phenotype with successive cytotoxic exposures.

Adaptive resistance mechanisms further constrain ICI activity in later lines of therapy, including upregulation of alternative immune checkpoint receptors (e.g., TIM-3, LAG-3, TIGIT), expansion of immunosuppressive myeloid-derived suppressor cell populations, and progressive stromal remodelling toward an immune-excluded phenotype [70].

Beyond PD-L1, an exploratory biomarker analysis from KEYNOTE-119 identified tumour mutational burden (TMB) as a potentially informative predictive variable. Among patients with TMB ≥10 mutations/Mb, a trend toward improved ORR and OS with pembrolizumab was observed, though patient numbers were small and findings were not formally hypothesis-tested [71]. This observation supports the routine use of comprehensive genomic profiling, including TMB quantification, in patients with metastatic TNBC, as a biologically distinct subset may retain sensitivity to checkpoint blockade irrespective of line of therapy. It should be noted, however, that high TMB (defined as ≥10 mutations/Mb) is uncommon in breast cancer overall, occurring in approximately 5% of cases, though is somewhat enriched in TNBC [72]. Tumour-agnostic FDA approval for pembrolizumab in TMB ≥10 solid tumours was based on KEYNOTE-158 pan-tumour data rather than randomised evidence in breast cancer specifically.

### 4.3 Early-Relapsing Disease

A clinically important and biologically distinct subgroup comprises patients who relapse early following prior curative-intent treatment. The phase III IMpassion132 trial (NCT03371017) enrolled patients with advanced TNBC relapsing within 12 months of completion of surgery or systemic therapy and randomly assigned them to first-line chemotherapy (carboplatin/gemcitabine or capecitabine) with or without atezolizumab [73]. This trial was conducted largely before (neo)adjuvant immunotherapy became standard practice. With a median follow-up of 9.8 months, no improvement was observed in the primary endpoint of OS in the PD-L1-positive population (median 12.1 versus 11.2 months; HR 0.93; 95% CI 0.73–1.20; *p* = 0.59), despite numerically higher objective response rates (40% versus 28%) and a longer median duration of response (6.6 versus 4.1 months) in the atezolizumab arm [73]. Median PFS was approximately 4 months across treatment arms, underscoring the generally treatment-refractory nature of this disease subset. These data indicate that early-relapsing TNBC represents an immune-resistant microenvironment, which significantly restricts the activity of current chemo-immunotherapy combination strategies.

Transcriptomic analyses have shed light on the underlying resistance biology. Compared with non-relapsing TNBC, both rapidly relapsing (rrTNBC) and late-relapsing (lrTNBC) tumours harbour substantially lower immune gene signatures, which correlate strongly with inferred anti-tumour immune cell populations including CD8^+^ T cells, M1 macrophages, and memory B-cells on CIBERSORT analysis [74]. Early-relapsing tumours are enriched for basal-like molecular features, carry a higher prevalence of *TP53* mutations, and are characterised by low baseline TIL density, impaired antigen presentation machinery, and interferon signalling pathway dysregulation‚ collectively consistent with primary immune resistance. Intriguingly, late-relapsing tumours show relative enrichment of luminal gene expression signatures, suggesting a biologically and immunologically distinct phenotype [74]. There remain limited prospective data to guide the use of ICIs in patients who have experienced rapid relapse following prior ICI-containing regimens, and this is an area of active clinical and translational investigation.

Given the limitations of PD-(L)1 monotherapy and chemo-immunotherapy in PD-L1-negative and early-relapsing disease, combinatorial strategies are under active investigation to broaden immunological benefit and circumvent known resistance mechanisms. Several mechanistically distinct approaches have demonstrated promising preliminary activity which will be discussed later in this review.

### 4.4 Safety Profile

The safety profile of ICI-based therapy in TNBC is broadly consistent with experience in other solid tumours. The addition of checkpoint inhibitors to chemotherapy increases the incidence of immune-related adverse events (irAEs), including thyroid dysfunction, immune-mediated hepatitis, colitis, pneumonitis, and dermatological toxicities. Across phase III studies, grade 3 treatment-related adverse events were more frequent in combination arms, though excess toxicity was predominantly immune-mediated rather than cytotoxic in nature [15,16].

## 5 Antibody-Drug Conjugates

### 5.1 Mechanism of Action and Rationale in TNBC

ADCs are designed to deliver cytotoxic small-molecule payloads selectively to tumour cells via a tumour-associated antigen-targeting monoclonal antibody. Each ADC comprises three functional elements: an antigen-targeting antibody, a cytotoxic payload, and a chemical linker that connects them and governs payload release kinetics. After binding to the target antigen on the tumour cell surface, receptor-mediated endocytosis internalises the ADC-antigen complex. Subsequent lysosomal processing releases the active payload intracellularly, where it induces DNA damage or disrupts the mitotic apparatus depending on its mechanism of action. The theoretical advantage over conventional cytotoxic chemotherapy lies in the selective delivery of high local payload concentrations within the tumour cell, though off target toxicity and acquired resistance remain clinically relevant limitations [75].

A critical pharmacological property that distinguishes modern ADCs from earlier iterations is the bystander killing effect. Agents employing membrane-permeable payloads release their payload intracellularly following linker cleavage, after which the membrane-permeable drug diffuses into adjacent tumour cells regardless of antigen expression status. This bystander effect allows ADCs to partially mitigate the impact of intra-tumoural antigen heterogeneity, a property of particular relevance in TNBC, which is characterised by marked genomic and proteomic heterogeneity [23,76,77].

Several biological features of TNBC provide a strong rationale for ADC-based therapeutic strategies. First, trophoblast cell-surface antigen 2 (Trop2), a transmembrane calcium-signal transducer encoded by the *TACSTD2* gene, is overexpressed in approximately 85% of TNBC tumours and is associated with increased tumour aggressiveness and inferior prognosis, rendering it a broadly applicable and clinically validated ADC target across the TNBC population [78]. Second, TNBC is defined by the absence of ER, PR, and HER2 overexpression/amplification, which precludes patients from established endocrine and HER2-directed therapy and makes exploitation of alternative surface antigens therapeutically imperative. Third, the recognition that a substantial proportion of historically HER2-negative TNBC tumours express low (immunohistochemistry [IHC] 1+ or 2+/*in situ* hybridisation [ISH]-negative) or ultralow (IHC >0 but <1+) levels of HER2 protein has opened an additional layer of targetability with the highly potent HER2-targeted ADC, trastuzumab deruxtecan (T-DXd) [18]. Finally, preclinical and clinical data increasingly support the capacity of Topo I inhibitor-based ADC payloads to induce immunogenic cell death (ICD), characterised by danger signal release, calreticulin surface exposure, and dendritic cell activation, thereby providing a mechanistic rationale for their combination with ICIs [79].

Over the past decade, ADCs have produced the most clinically meaningful survival improvements in heavily pretreated TNBC and given attrition between treatment lines in metastatic TNBC, where approximately half of patients do not receive second-line therapy, there is substantial interest in moving ADCs into the first line setting. Table 3 summarises the key clinical trials of ADCs in metastatic breast cancer.

**Table 3:** Summary of Key ADC Clinical Trials in Metastatic TNBC.

Agent	Target/Payload	Trial (NCT) Phase	Treatment Arms	Setting	PD-L1 Eligibility	Key Outcomes	Status
**Sacituzumab govitecan (SG)**	Trop2/SN-38	**ASCENT** (NCT02574455) III	SG vs. CT (eribulin, vinorelbine, capecitabine, gemcitabine)	≥2L mTNBC	Unselected	mPFS 5.6 vs. 1.7 m (HR 0.41); mOS 12.1 vs. 6.7 m (HR 0.48); ORR (35% vs. 5%)	FDA/EMA approved
		**ASCENT-03** (NCT05382299) III	SG vs. CT (paclitaxel, nab-paclitaxel, or gemcitabine/carboplatin)	1L mTNBC DFI ≥ 6 m	PD-L1-negative or ICI-ineligible	mPFS 9.7 vs. 6.9 m (HR 0.62); mOS 21.5 vs. 20.2 m (immature); ORR 48% vs. 46%	ESMO 2025, NEJM 2025
		**ASCENT-04/KEYNOTE-D19** (NCT05382286) III	SG + pembro vs. CT + pembro (paclitaxel, nab-paclitaxel, or gemcitabine/carboplatin)	1L mTNBC	PD-L1-positive (CPS ≥ 10)	mPFS 11.2 vs. 7.8 m (HR 0.65); mOS immature (HR 0.89); ORR 60% vs. 53%	ASCO 2025, NEJM 2026
**Datopotamab deruxtecan (Dato-DXd)**	Trop2/DXd	**TROPION-PanTumor01** (NCT03401385) I	Dato-DXd monotherapy	≥1L mTNBC	Unselected	ORR ~30%	Basis for further development
		**TROPION-Breast02** (NCT05374512) III	Dato-DXd vs. CT (paclitaxel or nab-paclitaxel if taxane-naïve/DFI >12 mo; carboplatin, eribulin, or capecitabine if prior taxane and DFI ≤12 mo)	1L mTNBC (Any DFI, <12 m ≤20%)	PD-L1-negative or ICI-ineligible	mPFS 10.8 vs. 5.6 m (HR 0.57); mOS 23.7 vs. 18.7 m (HR 0.79); ORR 62.5% vs. 29.3%	ESMO 2025
		**BEGONIA** (NCT03742102) Ib/II	Dato-DXd + durva	1L mTNBC	Unselected	ORR ~79% (TNBC cohort)	Supports phase III TROPION-Breast05
		**TROPION-Breast05** (NCT06103864) III	Dato-DXd + durva vs. CT (paclitaxel, nab-paclitaxel, or gemcitabine/carboplatin) + pembro	1L mTNBC	PD-L1-positive (CPS ≥10)	Ongoing	Ongoing
**ESG401 (OQY-3258)**	Trop2/SN-38	**Epitome-001** (NCT04892342) Ia/Ib	ESG401	1L and pretreated mTNBC	Unselected	1L TNBC ORR 85%; intracranial ORR 41% in HER2-neg (including HR+) BM cohort	ESMO 2024/ESMO Open 2025
**Sacituzumab tirumotecan (Sac-TMT, MK-2870/SKB264)**	Trop2/KL610023	**OptiTROP-Breast01** (NCT05347134) III	Sac-TMT vs. CT (eribulin, capecitabine, gemcitabine, vinorelbine)	≥2L mTNBC	Unselected	mPFS 6.7 vs. 2.5 m (HR 0.32); mOS NE vs. 9.4 m (HR 0.53); ORR 45.4% vs. 12.0%	Approved China
**Ladiratuzumab vedotin (LV)**	LIV-1/MMAE	**SGNLVA-001** (NCT01969643) I	LV	Pretreated mTNBC	Unselected	ORR 32%; mPFS 11.3 wks	Manageable toxicity
		**SGNLVA-002** (NCT03310957) Ib/II	LV + pembro	1L mTNBC	PD-L1-unselected (Part D: CPS < 10)	ORR ~54%	Early interim data
**Patritumab deruxtecan (HER3-DXd)**	HER3/DXd	NCT02980341 I/II	HER3-DXd	Pretreated mTNBC	PD-L1 unselected	Early activity	Ongoing
**Trastuzumab deruxtecan (T-DXd)**	HER2/DXd	**DESTINY-Breast04** (NCT03734029) III	T-DXd vs. CT (capecitabine, eribulin, vinorelbine, or gemcitabine)	Pretreated HER2-low mBC	Unselected	In TNBC cohort: mPFS 8.5m vs. 2.9 m (HR 0.46); mOS 18.2 vs. 8.3m (HR 0.48)	FDA/EMA approved HER2-low
		**DESTINY-Breast15** (NCT05950945) IIIb	T-DXd monotherapy	Pretreated HER2-low or HER2-0 mBC	Unselected	Ongoing	Ongoing
**Disitamab vedotin (RC48/DV)**	HER2/MMAE	**SGNDV-004** (NCT06157892) **Ib/II**	DV + tucatinib	Pretreated HER2-low or HER2-expressing mBC	Unselected	Ongoing	Ongoing

ADC, antibody-drug conjugate; BICR, blinded independent central review; BM, brain metastases; CPS, combined positive score; CT, chemotherapy; Dato-DXd, datopotamab deruxtecan; DFI, disease-free interval; DoR, duration of response; DV, disitamab vedotin; durva, durvalumab; HER, human epidermal growth factor receptor; HER3-DXd, patritumab deruxtecan; HR, hazard ratio; ICI, immune checkpoint inhibitor; LV, ladiratuzumab vedotin; mBC, metastatic breast cancer; MMAE, monomethyl auristatin E; mOS, median overall survival; mPFS, median progression-free survival; NE, not estimable; ORR, objective response rate; PD-L1, programmed death-ligand 1; pembro, pembrolizumab; Sac-TMT, sacituzumab tirumotecan; SN-38, active metabolite of irinotecan; T-DXd, trastuzumab deruxtecan; Topo I, topoisomerase I; TPC, treatment of physician’s choice; TNBC, triple-negative breast cancer; 1L, first line; 2L, second line.

### 5.2 Sacituzumab Govitecan

Sacituzumab govitecan (SG; Trodelvy) is a Trop2-directed ADC incorporating a humanised anti-Trop2 IgG1 monoclonal antibody conjugated to SN-38 via a proprietary pH-sensitive, hydrolysable carbonate linker at a drug-to-antibody ratio (DAR) of approximately 7.6. The relatively labile nature of the linker enables not only intracellular payload release following lysosomal processing, but also a degree of extracellular payload release within the tumour microenvironment, which contributes to bystander activity whilst simultaneously generating a modest systemic SN-38 exposure that may underlie some off-target toxicities, notably diarrhoea [80].

The ASCENT trial (NCT02574455) was a global, open-label, randomised phase III study that enrolled 468 patients with relapsed or refractory metastatic TNBC who had received at least two prior lines of systemic therapy, with at least one in the metastatic setting. Participants were randomised 1:1 to SG (10 mg/kg intravenously on days 1 and 8 of each 21-day cycle) or TPC single-agent chemotherapy (eribulin, vinorelbine, capecitabine, or gemcitabine). SG demonstrated a statistically significant and clinically meaningful improvement in both primary and secondary endpoints: median PFS was 5.6 versus 1.7 months (HR 0.41; 95% CI 0.32–0.52; *p* < 0.001) and median OS was 12.1 versus 6.7 months (HR 0.48; 95% CI 0.38–0.59; *p* < 0.001), with an ORR of 35% versus 5% in favour of SG. The PFS benefit was consistent across all predefined subgroups, including patients who had received prior ICI therapy [17]. On the basis of these results, SG received full FDA approval in April 2021 and EMA approval for patients with metastatic TNBC who have received at least two prior therapies.

Patients with stable CNS disease were included in ASCENT, although were not part of the primary endpoint population. Unfortunately, little benefit (ORR 3.0%, median PFS 2.8 months) was seen in these patients on sub-group analysis [81].

#### 5.2.1 First-Line Development Sacituzumab Govitecan: PD-L1-Negative/ICI-Ineligible

ASCENT-03 (NCT05382299) is a randomised, open-label, phase III trial designed to evaluate SG versus TPC chemotherapy as first-line therapy for patients with previously untreated, locally advanced unresectable or metastatic TNBC who are ineligible for ICI-based regimens. Ineligibility for ICI was defined as PD-L1 negativity or otherwise unsuitable for ICI due to autoimmune contraindications, organ transplantation, or other clinical reasons. Approximately 60% of metastatic TNBC patients fall into this category and currently lack any approved ICI option. An important eligibility criterion was a disease-free interval (DFI) of at least 6 months from the completion of curative-intent (neo)adjuvant therapy for patients with recurrent disease, thereby selecting a population with a less immediately chemotherapy-refractory phenotype. *De novo* stage IV disease was also eligible. The trial permitted crossover from the TPC arm to SG upon disease progression.

Primary results demonstrated a statistically significant improvement in PFS with SG versus TPC chemotherapy which was most commonly gemcitabine/carboplatin (median PFS 9.7 versus 6.9 months; HR 0.62; *p* < 0.001). OS data were immature at the time of primary analysis, and the high crossover rate (82% of patients in the chemotherapy arm who received subsequent therapy received SG) will inevitably complicate future OS interpretation. ORR was comparable between arms (48% versus 46%), but duration of response was substantially longer with SG (median DOR 12.2 versus 7.2 months). This trial represents the first prospective randomised evidence supporting an ADC over chemotherapy in the first-line PD-L1-negative metastatic TNBC population [19].

#### 5.2.2 First-Line Development Sacituzumab Govitecan: PD-L1-Positive

ASCENT-04/KEYNOTE-D19 (NCT05382286) was a global, randomised, phase III trial evaluating SG in combination with pembrolizumab versus pembrolizumab in combination with chemotherapy (paclitaxel, nab-paclitaxel, or gemcitabine plus carboplatin) as first-line therapy for patients with PD-L1-positive (CPS ≥ 10) locally advanced unresectable or metastatic TNBC. A total of 443 patients were randomised, and again, protocol-specified crossover to single-agent SG was permitted for patients in the chemotherapy arm upon disease progression.

Primary results demonstrated a statistically significant improvement in PFS with SG plus pembrolizumab versus chemotherapy plus pembrolizumab (median PFS 11.2 versus 7.8 months; HR 0.65; 95% CI 0.51–0.84; *p* < 0.001) at a median follow-up of 14 months. ORR (60% versus 53%) and median DOR (16.5 versus 9.2 months) also favoured the SG combination arm. Notably, the median PFS in the standard arm was shorter than the 9.7 months reported in Keynote-355. OS data were immature at the primary analysis, though an early directional trend in favour of SG plus pembrolizumab was noted despite crossover. The safety profile was consistent with the known profiles of individual agents; grade ≥3 treatment-emergent adverse events (TEAEs) occurred in 66% versus 62% of patients in the respective arms. These data support SG plus pembrolizumab as a potential new standard of care for first-line PD-L1-positive metastatic TNBC, though at the time of writing this article it has not yet been FDA approved [20].

### 5.3 Datopotamab Deruxtecan

Datopotamab Deruxtecan (Dato-DXd) is also a Trop2-directed ADC incorporating a humanised anti-Trop2 IgG1 monoclonal antibody conjugated via a stable tetrapeptide-based cleavable linker to the exatecan-derived Topo I inhibitor DXd, at a DAR of 4. Compared with SG, the tetrapeptide linker of Dato-DXd is characterised by greater plasma stability, resulting in reduced extracellular payload release and a lower rate of haematological and gastrointestinal toxicities [82]. However, the DXd payload class carries a distinct toxicity profile dominated by stomatitis/mucositis and interstitial lung disease (ILD), the latter representing the most clinically serious and potentially life-threatening class toxicity requiring active monitoring and prompt intervention when conjugated to a HER2-directed antibody [18]. In contrast, Dato-DXd is associated with a considerably lower rate of ILD but is more commonly complicated by mucositis requiring careful attention to mouth care and ocular toxicity, mostly commonly dry eyes.

The phase I TROPION-PanTumor01 trial (NCT03401385) established the Dato-DXd recommended phase II dose (RP2D) of 6 mg/kg on day 1 of each 21-day cycle and provided initial proof-of-concept data in metastatic TNBC, with single-agent ORR of approximately 30% in heavily pretreated patients, including those with prior platinum and ICI exposure [83].

#### 5.3.1 First-Line Development Datopotamab Deruxtecan: PD-L1-Negative/ICI-Ineligible

TROPION-Breast02 (NCT05374512) is a randomised, open-label, phase III trial comparing Dato-DXd with investigator’s choice of chemotherapy (paclitaxel or nab-paclitaxel if taxane-naïve/DFI > 12 months, or capecitabine, carboplatin, or eribulin monotherapy if prior taxane and DFI ≤ 12 months) as first-line therapy for patients with locally recurrent inoperable or metastatic TNBC for whom immunotherapy was not an option. Unlike ASCENT-03, TROPION-Breast02 did not impose a minimum DFI criterion, thereby enrolling a broader population that included patients with rapidly relapsing disease (DFI <12 months), who were capped at a maximum of 20% of the total enrolment. Patients with stable brain metastases were also eligible, an important distinction from ASCENT-03.

Median PFS was 10.8 months with Dato-DXd versus 5.6 months with chemotherapy (HR 0.57; 95% CI 0.47–0.69; *p* < 0.0001), and median OS was 23.7 versus 18.7 months (HR 0.79; 95% CI 0.64–0.98; *p* = 0.029), the latter representing the first statistically significant OS benefit demonstrated in first-line ICI-ineligible metastatic TNBC in a phase III setting. Notably, unlike ASCENT-03, crossover to the ADC was not permitted and combination chemotherapy was not allowed as the standard arm. ORR was 62.5% versus 29.3% and median DOR was 12.3 versus 7.1 months in favour of Dato-DXd [22]. The grade ≥3 treatment-related adverse event (TRAE) rate was similar between arms (33% versus 29%), and fewer patients discontinued Dato-DXd than chemotherapy due to TRAEs (4% versus 7%) [22]. TROPION-Breast02 is now considered a potential paradigm-shifting trial that may establish Dato-DXd as a new first line standard of care option in this population.

Cross-trial comparison of ASCENT-03 and TROPION-Breast02 is instructive but requires careful interpretation given important design differences. TROPION-Breast02 enrolled a higher-risk population by virtue of its DFI-unrestricted eligibility and inclusion of patients with brain metastases, factors likely associated with poorer prognosis. The chemotherapy backbone comparators also differed between trials, and the high crossover rate in ASCENT-03 may confound future OS data from that study. The distinct toxicity profiles of SG and Dato-DXd may also inform agent selection in individual patients.

#### 5.3.2 First-Line Development Datopotamab Deruxtecan: PD-L1-Positive

The BEGONIA trial (NCT03742102) is a phase Ib/II platform study evaluating Dato-DXd in combination with durvalumab (anti-PD-L1) as first line therapy for patients with locally advanced or metastatic TNBC. Early efficacy data from the TNBC cohort demonstrated an ORR of approximately 79%, with a durable response profile across both PD-L1-positive and PD-L1-negative subgroups [84]. These data established the mechanistic and clinical proof-of-concept for ADC plus ICI combinations and supported the initiation of the confirmatory phase III programme.

TROPION-Breast05 (NCT06103864) is a randomised phase III trial evaluating Dato-DXd with or without durvalumab versus chemotherapy plus durvalumab in patients with previously untreated PD-L1-positive mTNBC. This trial directly parallels the ASCENT-04 design but tests the Dato-DXd plus durvalumab combination against the current ICI-chemotherapy standard of care in the PD-L1-positive population. This trial is currently ongoing.

### 5.4 ESG401 (OQY-3258)

ESG401 (OQY-3258) is a novel Trop2-directed ADC comprising a humanised anti-Trop2 IgG1 monoclonal antibody conjugated to SN-38 via a proprietary serum-stable cleavable linker at a DAR of 8 given as an infusion on days 1, 8, 15 of a 28-day cycle. The key structural distinction from SG is the stability of the linker, which is designed to minimise premature extracellular payload release in circulation, thereby potentially improving the therapeutic index and, critically, enhancing CNS penetration [85].

Early-phase data from the phase Ia/Ib trial (NCT04892342) demonstrated in the first line metastatic TNBC cohort (n = 20), the confirmed ORR was 85%, with a disease control rate (DCR) of 100% and median PFS not yet reached, comparing favourably with BEGONIA and other first-line ADC combination benchmarks. Notably, no cases of ILD were reported across the entire phase I programme, which may reflect the linker stability of ESG401 compared with DXd-class ADCs. The haematological toxicity profile was consistent with SN-38-based agents, with the most common grade ≥3 treatment-related adverse events being neutropenia and leukopenia [86].

### 5.5 Sacituzumab Tirumotecan

Sacituzumab tirumotecan (sac-TMT; MK-2870/SKB264) is a next-generation Trop2-directed ADC that retains the same humanized anti-Trop2 monoclonal antibody scaffold used in SG but features a distinct linker-payload construct designed to enhance stability and pharmacologic properties. Instead of the unstable CL2A linker used in SG, sac-TMT employs a mesyl-sulfonyl-pyrimidine-CL2A-carbonate linker to conjugate KL610023, a belotecan-derived topoisomerase I inhibitor, at an average DAR of approximately 7.4. Belotecan itself is a synthetic camptothecin analogue that is chemically distinct from SN-38, the active metabolite of irinotecan used in SG. This refined linker-payload architecture is intended to improve plasma stability and intracellular release, optimising drug-to-antigen delivery and potentially delivering a differentiated toxicity and pharmacokinetic profile compared with SG [87,88]. Sac-TMT is administered intravenously every two weeks (Q2W) and has received marketing approval in China for adult patients with unresectable locally advanced or metastatic TNBC after at least two prior systemic therapies, and for EGFR-mutant non-small cell lung cancer (NSCLC), making it one of the first Trop2 ADCs approved in lung cancer.

The first-in-human phase I/II study (NCT04152499) established recommended dose of 4 mg/kg and 5 mg/kg Q2W in patients with advanced solid tumours refractory to standard therapies. In the phase II expansion TNBC cohort (n = 59), the ORR was 37% overall (35% at 4 mg/kg; 39% at 5 mg/kg), with a median DOR of 11.5 months and median PFS of 5.7 months, a durable response profile in heavily pretreated patients. Median OS was 15.7 months [88].

The Phase III OptiTROP-Breast01 trial (NCT05347134) conducted in China assessed sac-TMT versus TPC single-agent chemotherapy in patients with locally recurrent or metastatic TNBC who had received two or more prior therapies, including at least one for metastatic disease. The median PFS was 6.7 months (95% CI 5.5–8.0) with sac-TMT versus 2.5 months (95% CI 1.7–2.7) with TPC chemotherapy (HR 0.32; 95% CI 0.24–0.44; *p* < 0.00001). At the concurrent OS interim analysis, median OS was not reached (95% CI 11.2 months to NE) with sac-TMT versus 9.4 months (95% CI 8.5–11.7) with chemotherapy (HR 0.53; 95% CI 0.36–0.78; *p* = 0.0005). ORR was 45.4% versus 12.0% (*p* < 0.00001), and median DOR was 7.1 months (95% CI 5.6-NE) versus 3.0 months (95% CI 2.5-NE) in favour of sac-TMT. PFS benefit was consistent across predefined subgroups. The most common treatment-related adverse event with sac-TMT was haematological toxicity, and the overall safety profile was considered manageable [89].

OptiTROP-Breast05 (NCT05445908) is a phase II study evaluating sac-TMT at 5 mg/kg Q2W as first-line therapy for patients with advanced/metastatic TNBC (any PD-L1 status). Initial results presented at ASCO 2025 support activity in the 1L TNBC setting, with a signal of particular interest in the CPS < 10 (PD-L1-negative) population where current options are most limited [90]. Full efficacy data are awaited.

### 5.6 Ladiratuzumab Vedotin

Ladiratuzumab vedotin (LV; SGN-LIV1A) is an ADC directed against LIV-1 (SLC39A6), a zinc transporter and E-cadherin sheddase that is highly expressed in TNBC and implicated in epithelial-to-mesenchymal transition (EMT) and metastatic progression [91]. LV employs a protease-cleavable linker conjugated to monomethyl auristatin E (MMAE), a potent microtubule-disrupting agent with bystander killing capacity.

Phase I monotherapy data from SGNLVA-001 (NCT01969643) demonstrated single-agent activity in heavily pretreated mTNBC, with an ORR of 32%, CBR of 36%, and median PFS of 11.3 weeks [92]. The predominant toxicities reflected the MMAE payload class which include peripheral neuropathy, neutropenia, fatigue, and alopecia.

SGNLVA-002 (NCT03310957) is a single-arm, open-label phase Ib/II study evaluating LV in combination with pembrolizumab as first line therapy for patients with unresectable locally advanced or metastatic TNBC. The trial is PD-L1 unselected across most cohorts, with Part D specifically enrolling patients with CPS < 10 to address the PD-L1-negative population. Preliminary efficacy data from the combination cohort demonstrated an ORR of approximately 54% in first line metastatic TNBC at a minimum follow-up of 3 months, which compared favourably with chemotherapy-pembrolizumab benchmarks in unselected populations [93,94]. Confirmatory phase III development is under evaluation.

### 5.7 Patritumab Deruxtecan

Patritumab deruxtecan (HER3-DXd; U3-1402) is a HER3-directed ADC using the DXd payload and cleavable tetrapeptide linker shared with trastuzumab deruxtecan. HER3 is a pseudokinase receptor tyrosine kinase with the ability to dimerise with EGFR or HER2 and near-universal expression across breast cancer subtypes, including TNBC, with a potential role in acquired resistance to EGFR-targeted, HER2-directed, and cytotoxic therapy [95]. Unlike Trop2 or HER2, HER3 expression is independent of ER/PR/HER2 status, rendering it an attractive pan-subtype TNBC target. The bystander effect of DXd further mitigates the impact of intratumoural HER3 expression heterogeneity.

The phase I/II dose-escalation and expansion trial (NCT02980341) enrolled patients with multiple solid tumour types including metastatic TNBC. Early-phase data demonstrated single-agent activity in heavily pretreated TNBC patients, including those with prior Topo I inhibitor-based ADC exposure [96]. The capacity of patritumab deruxtecan to retain activity in post-SG and post-T-DXd settings is of particular clinical relevance given the likelihood that patients will have been exposed to earlier-line Topo I inhibitor-based ADCs as these agents assume earlier positions in the treatment algorithm. The safety profile is consistent with the DXd class, with ILD as the principal serious adverse event of interest.

The TUXEDO-3 trial demonstrated promising activity for HER3-DXd in patients with leptomeningeal disease, a rare but deadly complication of advanced breast cancer. Six of the 20 study patients had TNBC and the study met its primary endpoint of 3-month overall survival which was 65%. The intracranial response rate was 11.1%, with a 50% intracranial clinical benefit rate [97].

### 5.8 HER2-Low and HER2-Ultralow Disease

The dichotomous classification of breast cancer as HER2-positive (IHC 3+, or 2+/ISH+) or HER2-negative has been fundamentally challenged by the therapeutic validation of HER2-low disease as a distinct and actionable category. HER2-low is defined as IHC 1+ or IHC 2+/ISH-negative, which is a definition that captures approximately 45–55% of all breast cancers, including ~30% of TNBC tumours [98]. A further subset, designated HER2-ultralow (IHC >0 but <1+, with incomplete or faint membrane staining in any proportion of cells), may account for an additional 10–20% of breast cancers, though precise estimates vary considerably across studies given significant inter-observer variability in identifying this category [99]. The expansion of HER2-directed ADC eligibility to encompass these populations represents a potentially transformative development, bringing the vast majority of the HER2-negative breast cancer population, who were previously ineligible for any HER2-targeted therapy, within the scope of HER2-directed treatment.

#### 5.8.1 Trastuzumab Deruxtecan

Trastuzumab deruxtecan (T-DXd; Enhertu) is an anti-HER2 IgG1 ADC conjugated to DXd via a tetrapeptide cleavable linker at a DAR of 8. Its high DAR, membrane-permeable payload, and bystander effect enable it to exert antitumour activity at HER2 expression levels that are insufficient to support the efficacy of conventional HER2-targeted antibody or kinase-inhibitor therapies [18].

DESTINY-Breast04 (NCT03734029) was a global, randomised, phase III trial enrolling 557 patients with previously treated HER2-low (IHC1+ or 2+/ISH-) metastatic breast cancer who had received one to two prior lines of chemotherapy. Patients were randomised 2:1 to T-DXd (5.4 mg/kg Q3W) or TPC chemotherapy. The primary endpoint was PFS in the HR-positive cohort. Of the enrolled population, 58 patients (10%) had TNBC. In the overall population, T-DXd improved median PFS (9.9 versus 5.1 months; HR 0.50; 95% CI 0.40–0.63; *p* < 0.001) and median OS (23.4 versus 16.8 months; HR 0.64; 95% CI 0.49–0.84; *p* = 0.001). Although the TNBC subgroup was underpowered for definitive conclusions, the directional benefit was consistent with the overall population. The ILD rate was 12.1% (any grade) and 0.8% (grade ≥3), with close monitoring protocols mandated [18]. T-DXd received FDA and EMA approval for HER2-low metastatic breast cancer on the basis of DESTINY-Breast04 data.

DESTINY-Breast06 (NCT04494425) subsequently extended the HER2-targeting paradigm to the ultralow expression category in HR-positive metastatic breast cancer, demonstrating significant PFS improvement in the HER2-low population (HR 0.62; median 13.2 vs. 8.1 months; *p* < 0.0001) with directional benefit in the HER2-ultralow exploratory cohort (HR 0.78) [100], forming the basis for FDA expansion of the T-DXd indication to include HER2-ultralow HR-positive metastatic breast cancer in January 2025. Whether similar benefit extends to TNBC remains under active investigation. DESTINY-Breast15 (NCT05950945) is a phase IIIb study currently evaluating T-DXd in patients with HR-positive or HR-negative (including TNBC) HER2-low or HER2 IHC 0 metastatic breast cancer, which will provide the first prospective data on T-DXd activity across the full spectrum of HER2 expression in TNBC, including patients with no detectable HER2 expression [101].

#### 5.8.2 Disitamab Vedotin

Disitamab vedotin (RC48) is a HER2-directed antibody-drug conjugate (ADC) comprising a humanised anti-HER2 monoclonal antibody with enhanced binding affinity at lower levels of HER2 expression compared with trastuzumab-based constructs, conjugated to monomethyl auristatin E (MMAE) via a cleavable linker. This design enables activity across HER2-low and selected HER2-expressing tumours. Clinical studies evaluating disitamab vedotin in HER2-low or HER2-expressing metastatic breast cancer, including TNBC subgroups, have demonstrated preliminary antitumour activity, with a toxicity profile consistent with auristatin-based ADCs, notably peripheral neuropathy and neutropenia [102]. Disitamab vedotin may offer a complementary or alternative approach to T-DXd in HER2-low TNBC, particularly in contexts where DXd-class ILD risk represents a clinical concern. A number of clinical trials of monotherapy and combination strategies are underway in HER2 low and HER2-positive advanced breast cancer.

### 5.9 Safety and Tolerability Considerations of ADCs

The toxicity profiles of ADCs in metastatic TNBC are principally determined by the payload class, modified by the antibody component, and off-target antigen expression and linker stability also contribute. A practical understanding of payload-class toxicities is essential for patient selection, monitoring, and supportive care planning, particularly as patients may be sequentially exposed to multiple ADC agents over their treatment journey.

SN-38-based ADCs, exemplified by SG, are characterised predominantly by haematological toxicity (grade ≥3 neutropenia in approximately 50–65% of patients) and gastrointestinal toxicity. Diarrhoea with SG has a dual aetiology: early-onset cholinergic diarrhoea attributable to SN-38 and a late-onset secretory mechanism; grade ≥3 diarrhoea occurred in 10% of patients in ASCENT and requires proactive antidiarrhoeal prophylaxis. G-CSF prophylaxis is recommended per label. The rate of ILD with SG is very low (<1%), making it a preferable option in patients with underlying pulmonary comorbidities or prior pulmonary toxicity. Alopecia is expected [17].

DXd-based ADCs (T-DXd, Dato-DXd, patritumab deruxtecan) share a class-defining toxicity profile that prominently includes ILD/pneumonitis (especially T-DXd) and stomatitis/mucositis (especially Dato-DXd). ILD is the most serious toxicity and the leading cause of treatment-related fatality associated with this class; it occurs in approximately 10–15% of patients (any grade) with T-DXd and 5% with Dato-DXd, with grade ≥3 events in approximately 2% [103]. All patients initiating DXd-class therapy require mandatory pulmonary monitoring, patient education, and a low threshold for treatment interruption and prompt investigation of respiratory symptoms with high-resolution computed tomography (HRCT) [100]. Stomatitis/mucositis occurs in approximately 55% of patients at any grade with Dato-DXd [103] and again represents a class effect of the DXd payload, but is relatively uncommon with T-DXd and HER3-DXd. This is generally manageable with standard oral hygiene protocols and topical agents. Haematological toxicities are less prominent than with SN-38-based agents. The apparently lower ILD rate with ESG401, with no ILD demonstrated in the phase I trial, may reflect its stable linker design, however, this observation requires prospective validation in larger patient populations. Ocular toxicity appears particular to Dato-DXd.

MMAE-based ADCs (LV, disitamab vedotin) are associated with cumulative dose-dependent peripheral sensory neuropathy, the principal dose-limiting toxicity, along with neutropenia, fatigue, alopecia, and gastrointestinal effects. Peripheral neuropathy requires regular clinical assessment and dose modification per protocol to mitigate long-term morbidity [92].

## 6 PARP Inhibitors in Advanced TNBC

Poly(ADP-ribose) polymerase inhibitors (PARPi) represent the most clinically established molecularly targeted therapy in metastatic TNBC, with two agents, olaparib and talazoparib, holding regulatory approval (Table 4). Following the landmark OlympiAD and EMBRACA phase III trials, which demonstrated PFS superiority over TPC in germline *BRCA1/2*-mutated (gBRCA1/2mut) HER2-negative metastatic breast cancer, PARPi have become a standard-of-care option in this genetically defined population [104,105]. Neither trial reported a statistically significant overall survival benefit, at least in part due to crossover.

EMBRACA but not OlympiAD included patients with fully treated CNS disease and reported an enhanced benefit in that sub-group (HR 0.32) [105]. Case reports suggest olaparib also has useful CNS penetration and activity [106].

Critically, the TBCRC-048 trial has since expanded the actionable HRD landscape beyond germline *BRCA1/2*, establishing somatic *BRCA1/2* mutations and germline *PALB2* mutations as additional predictive biomarkers of olaparib sensitivity [32].

**Table 4:** PARP Inhibitor Trials in Advanced TNBC.

Agent	Trial (NCT) Phase	Treatment Arms	Setting	Patient Selection	Key outcomes	Status/Notes
**Olaparib**	**OlympiAD** (NCT02000622) III	Olaparib 300 mg BD vs. CT (capecitabine/eribulin/vinorelbine)	≤2 prior lines cytotoxic CT mBC^a^; HER2-	g*BRCA1/2*mut (~50% TNBC)	PFS 7.0 vs. 4.2 m (HR 0.58); OS 19.3 vs. 17.1 m (HR 0.90, ns); ORR 59.9% vs. 28.8%;	FDA approved 2018
	**TBCRC-048 Cohort 1** (NCT03188965) II	Olaparib 300 mg BD	≤2 prior lines cytotoxic CT mBC^b^; HER2-	g*PALB2*, g*BRIP1*, g*RAD51C/D*, g*ATM*, g*CHEK2*	g*PALB2* expansion cohort: PFS 9.6 m; ORR 75%; CBR 83%	g*PALB2* responses equivalent to g*BRCA1/2*; *ATM/CHEK2* no responses
	**TBCRC-048 Cohort 2** (NCT03188965) II	Olaparib 300 mg BD	≤2 prior lines cytotoxic CT mBC^b^; HER2-	s*BRCA1/2*mut	PFS 5.6 m; ORR 36.7%	First prospective evidence of PARPi sensitivity in s*BRCA1/2*
**Talazoparib**	**EMBRACA** (NCT01945775) III	Talazoparib 1 mg OD vs. CT (capecitabine/eribulin/vinorelbine)	≤3 prior lines cytotoxic CT mBC^c^; HER2-	g*BRCA1/2*mut	PFS 8.6 vs. 5.6 m (HR 0.54) OS 19.3 vs. 19.5 m (HR 0.85, ns) ORR 62.6% vs. 27.2%	FDA approved 2018
**Veliparib**	**BROCADE3** (NCT02163694) III	Veliparib + carbo/pac vs. pbo + carbo/pac	≤2 prior lines cytotoxic CT MBC^d^; HER2-	g*BRCA1/2*mut	PFS 14.5 vs. 12.6 m (HR 0.71) OS 32.4 vs. 28.2 m (HR 0.92, ns) ORR 75.8% vs. 74.1%	Not approved
**Olaparib + Pembrolizumab**	**KEYLYNK-009** (NCT04191135) II	Pembrolizumab + olaparib vs. pembrolizumab + CT (capecitabine, gemcitabine, or eribulin) (maintenance)	Post-induction 1L mTNBC (after pembro + platinum benefit)	CPS unselected; s*BRCA1/2* subgroup exploratory	PFS 5.5 vs. 5.6 m (HR 0.98, ns) OS 25.1 vs. 23.4 m (HR 0.95, ns)	Primary endpoint not met in unselected population s*BRCA1/2* subgroup PFS HR 0.70; OS HR 0.81

^a^OlympiAD: Prior anthracycline and taxane required unless contraindicated. HR+ patients required prior progression on at least one endocrine therapy (adjuvant or metastatic), unless endocrine therapy was considered clinically inappropriate. ^b^TBCRC-048: No requirement for prior anthracycline or taxane exposure. No limit on prior hormone, immune, or targeted therapies. ^c^EMBRACA: Prior anthracycline and/or taxane required unless contraindicated. No limit on number of prior hormone therapies. ^d^BROCADE3: No mandatory prior anthracycline or taxane requirement. HR+ patients eligible if deemed appropriate candidates for combination chemotherapy by the investigator. BD, twice daily; CBR, clinical benefit rate; carbo/pac, carboplatin/paclitaxel; CPS, combined positive score; CT, chemotherapy; gATM, germline ATM; gBRCA, germline BRCA; gBRIP1, germline BRIP1; gCHEK2, germline CHEK2; gPALB2, germline PALB2; gRAD51C/D, germline RAD51C/D; HER2−, HER2-negative; HR, hazard ratio; m, months; mBC, metastatic breast cancer; mOS, median overall survival; mPFS, median progression-free survival; NR, not reached; ns, not significant; OD, once daily; ORR, objective response rate; OS, overall survival; PARPi, PARP inhibitor; pbo, placebo; PFS, progression-free survival; sBRCA, somatic BRCA; TNBC, triple-negative breast cancer; TPC, treatment of physician’s choice; 1L, first line.

### 6.1 Mechanism of Action

PARP1 and PARP2 are pivotal mediators of single-strand DNA break (SSB) repair via the base excision repair (BER) pathway. PARPi exert cytotoxicity through two mechanisms: catalytic inhibition, preventing SSB repair, and PARP trapping, whereby the inhibitor stabilises the PARP-DNA complex, generating a protein-DNA adduct far more lethal than catalytic inhibition alone [107]. The degree of PARP trapping differs substantially across agents and correlates with clinical efficacy, talazoparib is among the most potent, while veliparib is comparatively weak [108,109]. In cells harbouring pathogenic variants in *BRCA1* or *BRCA2*, which are indispensable for homologous recombination (HR)-mediated DNA double-strand break (DSB) repair, unrepaired DSBs accumulate in the context of PARP inhibition, resulting in mitotic catastrophe and cell death. This principle of synthetic lethality underpins the selective efficacy of PARPi in HR-deficient tumours [110].

### 6.2 Key Clinical Trials of PARP inhibitors in TNBC

OlympiAD (NCT02000622) is a phase III trial that randomised 302 patients with HER2-negative g*BRCA1/2*mut metastatic breast cancer (50% TNBC) to olaparib 300 mg twice daily versus TPC (capecitabine, eribulin, or vinorelbine but not carboplatin). Olaparib significantly improved PFS (7.0 vs. 4.2 months; HR 0.58; 95% CI 0.43–0.80; *p* < 0.001) and ORR (59.9% vs. 28.8%). OS was numerically improved (19.3 vs. 17.1 months) but did not reach statistical significance [104,111].

The EMBRACA trial (NCT01945775) compared talazoparib with TPC in 431 patients with HER2-negative g*BRCA1/2*mut advanced breast cancer and demonstrated superior PFS (8.6 vs. 5.6 months; HR 0.54; 95% CI 0.41–0.71; *p* < 0.001), ORR (62.6% vs. 27.2%), and significantly better patient-reported outcomes. OS was not significantly different (19.3 vs. 19.5 months). Both olaparib and talazoparib are FDA-approved in this setting [105].

In the phase III BROCADE3 (NCT02163694) trial, veliparib was added to carboplatin and paclitaxel in 509 patients with g*BRCA1/2*mut HER2-negative advanced breast cancer. The triplet significantly prolonged PFS versus carboplatin/paclitaxel plus placebo (14.5 vs. 12.6 months; HR 0.71; *p* = 0.0016). OS was not significantly different at final analysis (32.4 vs. 28.2 months; HR 0.92; *p* = 0.43), and veliparib has not received regulatory approval in this setting [112].

TBCRC-048 is a landmark phase II single-arm trial that prospectively evaluated olaparib monotherapy in patients with HER2-negative advanced breast cancer harbouring somatic or germline HRR mutations beyond the established *gBRCA1/2* indication. The trial enrolled two distinct cohorts: Cohort 1 comprised patients with germline mutations in non-*BRCA1/2* HRR genes (g*PALB2*, g*BRIP1*, g*RAD51C/D*, g*ATM*, g*CHEK2*), and Cohort 2 comprised patients with somatic *BRCA1/2* mutations or somatic mutations in other HRR genes [32,113]. Among 27 patients in Cohort 1, the ORR was 33% overall. Critically, activity was strongly concentrated in g*PALB2*-mutated patients: in the expansion cohort of 24 patients with g*PALB2* mutations, the ORR was 75% (95% CI 53–90%) and CBR 83%, with a median PFS of 9.6 months, which approach the efficacy observed in studies *of* olaparib in g*BRCA1/2*mut disease [32,113]. By contrast, patients harbouring germline *ATM* or *CHEK2* mutations alone demonstrated no objective responses, establishing an important biomarker boundary within the germline HRR-mutant population. In the 27 patients in Cohort 2, the ORR in somatic *BRCA1/2*-mutated patients was 50% (95% CI 27–73%), with a median PFS of 6.3 months, demonstrating for the first time that somatic *BRCA1/2* mutations confer clinically meaningful sensitivity to PARPi, albeit with somewhat lower and shorter responses compared with germline carriers, although these differences should be interpreted cautiously given small patient numbers and potential biological heterogeneity. In the subsequent 2026 expansion cohort of 30 patients, the ORR was 36.7% and median PFS 5.6 months [113]. Among TNBC patients specifically in Cohort 2, 5 of 10 evaluable patients (50%) responded to olaparib, providing direct evidence of efficacy in the somatic *BRCA*-mutated TNBC subgroup [32].

TBCRC-048 substantially broadens the biological rationale for PARPi in advanced TNBC by demonstrating that somatic *BRCA1/2* mutations, identified through tumour-based next-generation sequencing (NGS) rather than germline testing, predict clinically meaningful sensitivity to olaparib. It also demonstrated that germline *PALB2* mutations confer a level of response equivalent to *gBRCA1/2*, supporting regulatory evaluation of PARPi in this population, but germline *ATM* and *CHEK2* mutations alone are insufficient to predict PARPi response in this biomarker-defined context. These findings provide a compelling rationale for incorporating somatic tumour profiling alongside germline testing in all patients with advanced TNBC being considered for PARPi, substantially expanding the fraction of eligible patients, estimated at an additional 5–8% of advanced TNBC above the *gBRCA1/2*-defined 15–20% [32].

The phase 2 KEYLYNK-009 trial (NCT04191135) evaluated pembrolizumab plus olaparib as postinduction maintenance therapy against continued pembrolizumab plus chemotherapy in patients with locally recurrent inoperable or metastatic TNBC who had derived clinical benefit from first-line pembrolizumab plus platinum-based chemotherapy induction. Among 271 patients randomised to postinduction therapy, the primary endpoint was not met, median PFS was 5.5 months in the pembrolizumab plus olaparib arm versus 5.6 months in the pembrolizumab plus chemotherapy arm (HR 0.98; 95% CI 0.72–1.33; *p* = 0.4556), with no difference in overall survival (25.1 vs. 23.4 months; HR 0.95; 95% CI 0.64–1.40). Importantly, however, a positive trend favouring pembrolizumab plus olaparib was observed in the somatic *BRCA1/2*-mutated subgroup for both PFS (HR 0.70; 95% CI 0.33–1.48) and OS (HR 0.81; 95% CI 0.28–2.37), suggesting a potential chemotherapy-free maintenance strategy warrants further investigation in biomarker-selected populations. Treatment-related adverse events were less frequent in the olaparib combination arm compared with the chemotherapy continuation arm (84.4% vs. 96.2%), highlighting a more favourable tolerability profile despite equivalent efficacy [114]. The negative result from KEYLYNK-009 in the unselected population underscores the critical importance of prospective biomarker-driven patient selection when combining PARP inhibition with immunotherapy in TNBC, and reinforces that the preclinical synergy between these agents does not translate uniformly across an unselected patient population.

## 7 HER2 (ERBB2) Mutations in HER2 Non-Amplified TNBC

*HER2* missense mutations occur in approximately 2–5% of TNBC in the absence of gene amplification, and result in ligand-independent activation of HER2 signalling pathways, promoting oncogenic growth and survival. Standard anti-HER2 monoclonal antibodies are largely ineffective against kinase-domain mutant HER2, whereas HER2-targeted strategies incorporating irreversible tyrosine kinase inhibitors (TKIs) such as neratinib and dual HER2-directed combinations such as tucatinib and trastuzumab, retain activity against this molecularly distinct subgroup.

SUMMIT (NCT01953926) is a phase II basket trial evaluating neratinib in HER2-mutant cancers. In the HR-positive, HER2-mutant metastatic breast cancer cohort, the neratinib plus fulvestrant plus trastuzumab triplet demonstrated an ORR of 40%, leading to NCCN recognition as a treatment option for HER2-mutated metastatic breast cancer. In the TNBC-specific population, ORR with neratinib-based combinations was approximately 33.3% and median PFS was 6.2 months [115,116].

The plasmaMATCH trial (NCT03182634) was a multicentre, phase 2a, ctDNA-directed precision oncology platform in patients with advanced breast cancer, designed to match targeted therapies to actionable mutations identified in circulating tumour DNA. In this trial, patients with activating HER2 mutations detected by ctDNA profiling were assigned to a treatment cohort of neratinib monotherapy for hormone receptor-negative disease or neratinib plus fulvestrant for HR-positive disease. Among the 20 efficacy-evaluable participants with HER2 mutations, ~25% achieved a confirmed objective response, and additional unconfirmed responses were seen, with a median PFS of approximately 5.4 months and a median duration of response of 5.7 months [30]. The plasmaMATCH results demonstrated that ctDNA-based screening could effectively identify patients with rare HER2 mutations and guide targeted therapy, providing clinical evidence that HER2 mutations are actionable in metastatic breast cancer. However, responses were less pronounced in patients without concomitant endocrine targeting, highlighting the heterogeneity of HER2 mutation biology.

SGNTUC-019 (NCT04579380) was a phase 2 basket study evaluating the combination of the HER2-selective tyrosine kinase inhibitor tucatinib plus trastuzumab (+/- fulvestrant) in patients with previously treated, metastatic solid tumours harbouring activating *HER2* mutations, including a dedicated cohort of 29 patients with HER2-mutated metastatic breast cancer that was HER2-negative by local testing and included 4 patients with TNBC. In the HER2-mutated metastatic breast cancer cohort, tucatinib plus trastuzumab (+/- fulvestrant) demonstrated clinically meaningful antitumour activity, with a confirmed ORR of approximately 41.9%, DCR of 80.6%, and median OS 20.1 months, supporting the concept of targeting *HER2* mutations even in the absence of gene amplification [35].

These data provide prospective evidence supporting dual HER2-targeted therapy in HER2-mutant, HER2-non-amplified breast cancer and highlight the importance of precision HER2 inhibition beyond traditional overexpression/amplification paradigms.

T-DXd has also been evaluated in *HER2*-mutated disease in the DESTINY-PanTumour01 trial (NCT04639219), a basket study of 102 patients with previously treated advanced solid tumours including 20 with breast cancer [37]. Although the ORR in the study was 29.4%, ORR in the small breast cancer subgroup was 50%.

Importantly, neratinib, tucatinib-based regimens, and trastuzumab deruxtecan have demonstrated intracranial activity in HER2-positive breast cancer, providing a rationale for their evaluation in HER2-mutated disease [117,118,119].

## 8 Other Emerging Targeted Therapies in Advanced TNBC

Beyond PARPi, the therapeutic landscape for advanced TNBC is increasingly shaped by a range of molecularly targeted strategies in various stages of clinical development, each exploiting distinct oncogenic dependencies or vulnerabilities within defined biomarker-selected subpopulations. These include PI3K/AKT/mTOR pathway inhibitors, androgen receptor antagonists, and FGFR inhibitors. Biomarker-driven patient selection is a unifying theme across all these strategies. Table 5 summarises the key clinical trials of targeted therapies in metastatic breast cancer.

**Table 5:** Targeted Therapies in Advanced TNBC.

Target/Class	Agent	Trial (NCT) Phase	Treatment Arms	Setting/Line	Patient Selection	Key Outcomes	Status
**PI3K/AKT/mTOR**	**Ipatasertib**	**IPATunity130 Cohort A** (NCT03337724) III	Ipatasertib + paclitaxel vs. pbo + paclitaxel	1L mTNBC	PIK3CA/AKT1/PTEN-altered	PFS 7.4 vs. 6.1 m (HR 1.02; ns); ORR 39% vs. 35%; OS 24.4 vs. 24.9 m (HR 1.08; ns)	Negative; no benefit demonstrated in enriched population
		**CO40151** (NCT03800836) Ib	Atezolizumab + ipatasertib + paclitaxel	1L mTNBC	PIK3CA/AKT1/PTEN-unselected	ORR 73% (early cohort, n = 26)	Early phase signal
	**Capivasertib**	**PAKT** (NCT02423603) II	Capivasertib + paclitaxel vs. pbo + paclitaxel	1L mTNBC	TNBC overall; PIK3CA/AKT1/PTEN-altered subgroup	**Overall:** PFS 5.9 vs. 4.2 m (HR 0.74), ORR 59% vs. 40%. **Altered subgroup:** PFS 9.0 vs. 4.9 m (HR 0.59), OS 19.1 vs. 12.6 m (HR 0.61)	Phase II proof-of-concept in altered subgroup
		**CAPItello-290** (NCT03997123) III	Capivasertib + paclitaxel vs. pbo + paclitaxel	1L mTNBC	TNBC overall; PIK3CA/AKT1/PTEN-altered subgroup	**Overall:** PFS 5.6 vs. 5.1 m (HR 0.72); ORR 50.1% vs. 37.6%; OS 17.7 vs. 18.0 m (HR 0.92; ns). **Altered subgroup:** PFS 7.5 vs. 5.6 m (HR 0.70); OS 20.4 vs. 20.4 m (HR 1.05; ns)	Dual OS primary endpoint not met in overall or altered subgroups; not approved
	**ALTA2618**	**AKTive-001** (NCT06533059) I/Ib	ALTA2618 monotherapy	Advanced solid pretreated tumours incl. mTNBC	AKT1 E17K mutant	Ongoing	Phase I dose escalation ongoing
**Androgen Receptor (AR)**	Bicalutamide	**TBCRC-011** (NCT00468715) II	Bicalutamide 150 mg OD	mTNBC (pretreated)	AR+ (≥10%) TNBC	PFS 12 wks; ORR 0%, CBR 19% at 6 months	Proof-of-concept for AR targeting
	Enzalutamide	**MDV3100-11** (NCT01889238) II	Enzalutamide 160 mg OD	mTNBC (pretreated)	AR+ (>0%) TNBC	**Overall:** PFS 2.9 m; CBR 25% at 16 wks; **AR ≥10% subgroup:** PFS 3.4 m; ORR 8%, CBR 33%; OS 17.6 m	LAR subtype enriched for benefit
	Abiraterone	**UCBG 12-1** (NCT01842321) II	Abiraterone acetate 1000 mg OD + prednisone 5 mg BD	mTNBC (pretreated)	AR+ (≥10%) TNBC	PFS 2.8 m; ORR 6.7%; CBR 20% at 6 months	Modest single-agent activity
**HER2 Mutations**	Neratinib ± fulvestrant ± trastuzumab	**SUMMIT** (NCT01953926) II	Neratinib ± fulvestrant ± trastuzumab	mBC incl. TNBC (pretreated)	HER2-mutant non-amplified	**TNBC:** PFS 6.2 m; ORR 33.3%; **HR+/HER2-mut:** PFS 8.3 m; ORR 39%	NCCN guideline inclusion for HER2-mut mBC (particularly HR+)
	Tucatinib + trastuzumab	**SGNTUC-019** (NCT04579380) II	Tucatinib + trastuzumab ± fulvestrant	mBC incl. TNBC (pretreated)	HER2-mutant non-amplified	ORR 41.9%; OS 20.1 m	Activity in HER2-mutant non-amplified disease
	Neratinib	**plasmaMATCH** (NCT03182634) II	Neratinib ± fulvestrant	mBC incl. TNBC (pretreated)	HER2 mutation detected by ctDNA	PFS 5.4 m; ORR 25%	ctDNA-directed patient selection
	T-DXd	**DESTINY-PanTumor01** (NCT04639219) II	T-DXd monotherapy	Previously treated advanced solid tumours incl. mTNBC	HER2-expressing or HER2-mutant non-amplified	**Breast cancer subgroup:** ORR 50%; Overall: ORR 29.4%	Demonstrates activity across HER2-driven solid tumours
**FGFR Mutations**	Futibatinib	**FOENIX-MBC2** (NCT04024436) II	Futibatinib monotherapy	mBC incl. TNBC	FGFR1–4 alterations (incl. FGFR2 amplification)	TNBC: ORR ~9.5%, PFS ~1.9 mo; HR+/HER2−: ORR ~18%, PFS ~7.2 mo	Modest activity overall; heterogeneous responses across subtypes

mBC, metastatic breast cancer; mTNBC, metastatic triple-negative breast cancer; TNBC, triple-negative breast cancer; HR+, hormone receptor–positive; HER2−, HER2-negative; ORR, objective response rate; CBR, clinical benefit rate; PFS, progression-free survival; OS, overall survival; ctDNA, circulating tumour DNA; ns, not significant; mo, months.

### 8.1 Androgen Receptor Targeting

Androgen receptor (AR) expression is identified in 10–35% of TNBC tumours, correlating closely with the LAR intrinsic subtype, characterised by high AR and downstream androgen-regulated gene expression, frequent *PIK3CA* alterations, and a relatively lower proliferative index compared with other TNBC subtypes [23,25,120]. Preclinical data robustly demonstrate AR-driven proliferation and survival in LAR tumour cells, providing the mechanistic rationale for androgen receptor antagonism.

TBCRC-011 (NCT00468715) is a phase II trial that evaluated Bicalutamide 150 mg daily in patients with AR-positive (≥10%) metastatic TNBC (n = 26). CBR at 6 months was 19% (95% CI 7–39%), median PFS was 12 weeks, and there were no objective responses. This study provided the first prospective clinical evidence supporting AR targeting in molecularly selected TNBC [121]. MDV3100-11 (NCT01889238) evaluated Enzalutamide in the same population and demonstrated a CBR of 25% at 16 weeks, ORR 8%, and median PFS 3.4 months in the evaluable AR ≥10% subgroup. Retrospective transcriptomic analysis suggested enrichment of benefit within the LAR subtype [38]. Abiraterone combined with prednisone showed minimal benefit in a phase 2 study of AR+ TNBC, with a reported response rate of 6.7% and 6-month CBR of 20% [39]. Darolutamide failed to demonstrate superiority over capecitabine in a phase 2 study of AR+ TNBC, highlighting ongoing challenges in identifying optimal biomarkers for AR-directed therapy [40].

The PAveMenT trial (NCT04360941) is a Phase Ib, open-label study evaluating the safety, tolerability, and preliminary efficacy of the CDK4/6 inhibitor palbociclib in combination with the PD-L1 ICI avelumab in patients with metastatic AR-positive TNBC, again selected by IHC. The trial incorporates a dose-escalation phase followed by an expansion cohort to evaluate preliminary efficacy in AR-positive TNBC. This combination strategy is based on preclinical rationale that CDK4/6 inhibition may enhance anti-tumour immunity and potentiate responses to PD-L1 blockade in molecularly defined TNBC subsets. The study is ongoing, with exploratory analyses evaluating AR expression, PAM50 subtype, PD-L1 status, and tumour-infiltrating lymphocytes as potential predictive biomarkers [122].

### 8.2 PI3K/AKT/mTOR Pathway Inhibition

#### 8.2.1 Rationale and Preclinical Evidence

The PI3K/AKT/mTOR axis is dysregulated in approximately 25–30% of TNBC tumours through activating mutations in *PIK3CA*, loss-of-function alterations in *PTEN*, and activating mutations in *AKT1* [123]. Selective inhibition of AKT with small-molecule inhibitors such as capivasertib and ipatasertib has demonstrated preclinical activity in TNBC models characterised by PI3K pathway dysregulation, including suppression of tumour cell survival and enhancement of the cytotoxic effects of taxane-based chemotherapy. These findings support the concept that targeting AKT, a convergent therapeutic node downstream of PI3K that can circumvent redundancy in upstream PI3K isoforms and feedback reactivation of RTKs, may help overcome intrinsic chemotherapy resistance in TNBC characterised by PI3K/AKT/mTOR pathway activation [124].

#### 8.2.2 Key Clinical Trials

IPATunity130 (NCT03337724) was a randomised phase III trial which evaluated the AKT inhibitor Ipatasertib plus paclitaxel versus placebo plus paclitaxel as first-line therapy for *PIK3CA/AKT1/PTEN*-altered advanced TNBC in Cohort A. Despite the positive phase II data from the LOTUS trial, the addition of Ipatasertib to paclitaxel did not improve PFS compared with placebo plus paclitaxel (median 7.4 months vs. 6.1 months; HR 1.02), and no meaningful OS benefit was observed, highlighting challenges in translating AKT inhibition into broad clinical benefit in molecularly defined TNBC [125].

The phase Ib CO40151 study (NCT03800836) evaluated first-line atezolizumab, ipatasertib, and taxane chemotherapy in metastatic TNBC. Among the first 26 evaluable patients, the confirmed ORR was 73%, with responses observed irrespective of PD-L1 status or PI3K pathway alterations. Although these findings suggested potential synergy between AKT inhibition and ICI, the small, non-randomised cohort limits definitive conclusions [126]. Subsequent phase III trials have not consistently reproduced this magnitude of benefit, highlighting the complexity of integrating AKT inhibition into chemo-immunotherapy backbones in TNBC.

The AKT inhibitor Capivasertib was evaluated in combination with paclitaxel versus placebo plus paclitaxel in first-line metastatic TNBC in the Phase II PAKT trial (NCT02423603). The overall PFS result was borderline (5.9 vs. 4.2 months; HR 0.74; *p* = 0.06), but the *PIK3CA/AKT1/PTEN*-altered subgroup demonstrated a pronounced PFS benefit (HR 0.59) and a significant OS advantage (19.1 vs. 12.6 months; HR 0.61; *p* = 0.035), providing proof-of-concept for biomarker-selected enrichment [127].

This led to the phase III CAPItello-290 trial (NCT03997123) which randomised 812 patients with previously untreated metastatic TNBC to capivasertib plus paclitaxel versus placebo plus paclitaxel. PFS numerically favoured capivasertib in both the overall population (5.6 vs. 5.1 months; HR 0.72; 95% CI 0.61–0.84) and the biomarker-altered (*PIK3CA/AKT1/PTEN*-altered) subgroup (7.5 vs. 5.6 months; HR 0.70; 95% CI 0.52–0.95), however the dual primary endpoint of OS was not met in either population, and capivasertib did not receive regulatory approval in this setting [128].

Capivasertib monotherapy was also evaluated in patients with *AKT* or *PTEN* mutations and TNBC in the PlasmaMATCH trial, however the cohort failed to meet its predefined efficacy threshold [30].

ALTA2618 is a covalent inhibitor designed specifically to target the *AKT1* E17K activating mutation which is present in approximately 2–5% of TNBC. Unlike pan-AKT inhibitors, its mutation selectivity is intended to improve the therapeutic index by sparing wild-type *AKT* signalling. Early phase I/II data from AKTive-001 trial (NCT06533059) demonstrate objective responses in *AKT1* E17K-mutant tumours, including TNBC, with a favourable tolerability profile [129].

### 8.3 FGFR Mutations

*FGFR* genomic alterations, encompassing *FGFR1* amplification, F*GFR2* amplification/activating mutations, and *FGFR3* fusions, activate downstream RAS/MAPK and PI3K/AKT signalling, promoting proliferation, angiogenesis, and epithelial-mesenchymal transition. Preclinical studies demonstrate selective FGFR inhibitors potently suppress *FGFR*-altered TNBC models, with synergistic activity in combination with chemotherapy in xenograft experiments [130].

Dovitinib (TKI258) was among the first FGFR inhibitors evaluated clinically in breast cancer [34], although subsequent development focused primarily on HR-positive disease rather than TNBC.

The FOENIX-MBC2 trial (NCT04024436) is a phase II study evaluating futibatinib across multiple cohorts of advanced breast cancer harbouring *FGFR* amplifications. While detailed subgroup results specific to TNBC have not yet been fully published, preliminary reports suggest that among TNBC patients with *FGFR* alterations treated with futibatinib, the ORR is 9.5%, with a DCR of 33% and a median PFS of approximately 1.9 months [131]. These findings highlight the importance of comprehensive NGS-based tumour profiling to identify FGFR-driven subsets and to enrich future targeted trials.

## 9 Emerging and Investigational Approaches

Beyond molecularly targeted strategies, a diverse array of investigational modalities is redefining the frontier of TNBC therapeutics, exploiting novel immune checkpoints, engineering improved tumour-directed delivery platforms, harnessing cell-based immunotherapy, and dissecting the complex immunosuppressive TME. Durable responses in advanced TNBC will likely require combinatorial strategies simultaneously engaging tumour-intrinsic vulnerabilities and reshaping the immune landscape. Furthermore, with the move of pembrolizumab into early TNBC and the potential for ADCs to become standard in the post-neo-adjuvant setting if TROPION-Breast03 and ASCENT-05 are positive, patients with disease relapsing despite these key agents will be increasingly difficult to treat and novel strategies will be urgently required.

### 9.1 Novel Checkpoints beyond PD-1/PD-L1: LAG-3, TIGIT, and VISTA

Despite the paradigm-shifting OS benefit of pembrolizumab combined with chemotherapy in PD-L1-positive (CPS ≥10) metastatic TNBC, a substantial proportion of patients do not respond or develop acquired resistance. Lymphocyte-activation gene-3 (LAG-3), which suppresses T cell activation through MHC class II binding on exhausted CD8^+^ T cells and Tregs, is one of the most clinically advanced alternative checkpoints. The combination of relatlimab (anti-LAG-3) with nivolumab achieved regulatory approval in melanoma (RELATIVITY-047) [132].

TIGIT (T cell immunoreceptor with Ig and ITIM domains) is an inhibitory checkpoint expressed on T cells and NK cells in TNBC, where it competes with CD226 for CD155 binding, suppressing antitumor immunity. Early-phase trials of the anti-TIGIT antibody Tiragolumab combined with PD-L1 inhibition and chemotherapy have demonstrated encouraging activity in TNBC, supporting further evaluation [133].

VISTA (V-domain Ig suppressor of T-cell activation) is predominantly expressed on myeloid cells within the TNBC microenvironment and represents a distinct inhibitory axis separate from PD-1/PD-L1. Translational analyses have shown that VISTA is upregulated in human TNBC tissues, where it is primarily expressed on tumour-infiltrating macrophages and neutrophils, and that VISTA blockade re-programs macrophages toward a pro-inflammatory phenotype and enhances CD8^+^ T-cell activation in preclinical models. These findings provide a mechanistic rationale for targeting VISTA in TNBC, although clinical efficacy data from VISTA antagonists in TNBC are still emerging [134].

### 9.2 Tumour-Associated Antigen Vaccines

Peptide and neoantigen-based vaccines represent a promising immunotherapeutic strategy in TNBC. GP2, a HER2-derived peptide vaccine administered with GM-CSF, demonstrated encouraging disease-free survival outcomes in a phase II trial of HER2 3+ patients with residual disease post-neoadjuvant chemotherapy, with exploratory signals suggesting activity in TNBC subgroups [135]. Personalised neoantigen vaccines, designed based on tumour mutational burden and whole-exome sequencing, are under investigation in combination with PD-1/PD-L1 blockade, aiming to convert immunologically “cold” TNBC tumours into “hot” phenotypes responsive to immune attack [136]. Additional vaccine targets include α-lactalbumin, re-expressed in basal-like TNBC, and AE37 (Ii-Key/HER2 peptide conjugate), which leverage tumour-specific antigens to stimulate cytotoxic T-cell responses [137,138]. These approaches highlight the potential of tumour-associated antigen vaccines to enhance immunogenicity and complement checkpoint inhibition in TNBC.

### 9.3 Novel ADCs and Targeted Delivery Platforms

Dual-payload ADCs couple two distinct cytotoxic warheads to circumvent resistance by attacking multiple cellular vulnerabilities. Immune-engaging ADCs combine cytotoxic payloads with immunostimulatory cargoes (TLR agonists, STING agonists) to convert focal cytotoxicity into systemic immune priming. TLR7/8 and STING agonist strategies have clear preclinical rationale in TNBC immunomodulation, with demonstrated ability in models to enhance innate and adaptive immune responses [139,140]. However, clinical evidence in TNBC is still early or emerging, with most studies to date confined to preclinical models or early phase trials in broader oncology indications rather than TNBC-specific cohorts.

Bicycle Toxin Conjugates (BTCs) represent a next-generation targeted delivery scaffold distinct from traditional ADCs. BTCs are built on ultrasmall (~1.5–2 kDa) conformationally constrained bicyclic peptides engineered for high-affinity target binding with favourable tissue penetration, rapid renal clearance from normal tissues, and scalable chemical synthesis. These properties result in rapid tumour targeting and systemic clearance, a pharmacokinetic profile distinct from conventional ADCs. BTCs carry potent cytotoxic warheads such as auristatin payloads via cleavable linkers to elicit direct tumour cell kill [141].

BT5528 (Zelenectide Pevedotin) targets the receptor tyrosine kinase EphA2 which is frequently overexpressed in TNBC and associated with aggressive biology, metastatic potential, and poor prognosis. In a phase I/II first-in-human study of BT5528 in patients with advanced solid tumours, emerging clinical data demonstrated a differentiated safety profile and preliminary antitumour activity across a heterogeneous population, with an ORR of 12% and generally low rates of peripheral neuropathy and other toxicities typically attributed to ADC payloads [142].

The Duravelo programme encompasses BTCs targeting multiple antigens. BT8009, a NECTIN4-targeting BTC, has been evaluated in phase I/II trials and received FDA Fast Track designation for previously treated locally advanced or metastatic solid tumours expressing NECTIN4 [143]. While TNBC-specific efficacy results are still emerging, these BTCs demonstrate promising safety and early activity signals, highlighting their potential as novel targeted therapeutics in TNBC and other solid tumours.

### 9.4 Cell-Based Therapies: CAR-T and CAR-NK Cells

Chimeric antigen receptor (CAR)-engineered adoptive cell therapies have garnered interest in TNBC. Multiple CAR-T programmes targeting tumour-associated antigens such as ROR1, MUC1, mesothelin, GD2, and EGFR have been explored preclinically and in early clinical studies, reflecting the heterogeneous antigen landscape of TNBC and the challenge of identifying robust, tumour-restricted targets. Preclinical models demonstrate potent antitumour cytotoxicity, and early clinical efforts suggest potential signals of activity, but overall ORR in solid tumour CAR-T trials remain modest compared with haematological malignancies, largely due to antigen heterogeneity, limited tumour trafficking and persistence, and immunosuppressive tumour microenvironments. These barriers, characterised by abundant regulatory T cells, myeloid-derived suppressor cells, and inhibitory cytokines such as TGF-β, constrain intra-tumoural CAR-T expansion and effector function and contribute to toxicity such as cytokine release syndrome [144].

CAR-NK cells offer a complementary adoptive platform with distinct advantages. These include MHC-unrestricted cytotoxicity, a lower propensity for graft-versus-host disease (GvHD), and the feasibility of “off-the-shelf” allogeneic products. CAR-NK constructs targeting antigens such as NKG2D ligands or CD44v6 have demonstrated appreciable cytotoxicity in TNBC preclinical models, including 3D tumour spheroid systems that better mimic the tumour microenvironment. Incorporation of cytokine support or engineering to overcome inhibitory signals has further enhanced CAR-NK potency *in vitro* [145].

Armoured CAR designs such as CAR-T cells co-expressing IL-15 or IL-12, TGF-β-resistant receptors, or dual-antigen constructs are being actively investigated to mitigate immunosuppression and antigen escape. Combining CAR-T or CAR-NK therapies with ICI, cytokines, or targeted agents is also under active study to enhance persistence and antitumour efficacy [146].

Despite encouraging preclinical evidence and early feasibility data, clinical efficacy in TNBC remains limited, with few durable complete responses reported to date. Ongoing early-phase clinical trials of CAR-T cells against novel targets such as mesothelin and EGFR in TNBC are underway, aiming to define safety, optimal dosing, and preliminary efficacy, while next-generation constructs continue to evolve to overcome tumour microenvironment barriers and improve patient outcomes [147].

### 9.5 Tumour Microenvironment-Targeted Therapies

#### 9.5.1 Macrophage Modulators and T-Cell Immunostimulants

The TNBC TME is enriched with immunosuppressive M2-polarised tumour-associated macrophages (TAMs) that promote immune evasion, angiogenesis, and metastasis. TAMs can be depleted or reprogrammed towards pro-inflammatory M1 phenotypes using CSF1R inhibitors such as Pexidartinib, which reduce macrophage recruitment and suppressive signalling in preclinical TNBC models, enhancing T-cell and B-cell infiltration and antitumour activity. Early clinical studies of CSF1R blockade in combination with ICI or chemotherapy demonstrate modulation of the immune milieu and target TAM-mediated suppression, supporting broader investigation [148].

#### 9.5.2 Stromal Barriers and TGF-β Inhibition

Desmoplastic stroma driven by cancer-associated fibroblasts (CAFs), extracellular matrix deposition, and TGF-β signalling forms a physical and biochemical barrier to effective immune cell infiltration and drug penetration. CAF subtypes such as FAP^+^ and CAF-S1 subsets contribute to immunosuppression and exclude effector T cells in TNBC [149]. Dual inhibition approaches targeting both the TME and checkpoint pathways have been pursued. Bintrafusp alfa, a bifunctional fusion protein that sequesters TGF-β while blocking PD-L1, has been evaluated in early-phase TNBC clinical trials (e.g., NCT04489940), however, trials were discontinued due to limited efficacy [150]. Galunisertib, a TGF-β receptor I kinase inhibitor, has also been investigated in combination regimens to counteract CAF-driven immunosuppression and enhance checkpoint blockade efficacy [151]. Additional strategies such as FAP-targeted CAR-T cells and bispecific antibodies aim to disrupt stromal components directly. These TME-reprogramming approaches emphasise that effective immunotherapy for advanced TNBC may require concurrent targeting of stromal, myeloid, and immune checkpoints [152].

## 10 Clinical Considerations, Challenges, and Unmet Needs

### 10.1 Treatment Sequencing in Metastatic TNBC

The evolving treatment landscape of metastatic TNBC is increasingly underpinned by a personalised medicine framework in which therapeutic selection is guided by prospectively assessed biomarkers rather than a uniform sequential chemotherapy approach. In clinical practice, PD-L1 status (CPS ≥10 by 22C3 or IC ≥1% by SP142), germline *BRCA1/2* and *PALB2* mutation status, and HER2 low/ultralow expression now define distinct actionable subpopulations with specific approved therapies. The integration of ctDNA monitoring at progression offers an additional dynamic layer of personalisation, enabling early identification of resistance mechanisms such as *BRCA* reversion mutations that inform subsequent treatment selection. This biomarker-stratified approach not only optimises therapeutic efficacy but also aims to reduce unnecessary toxicity by reserving agents with specific adverse event profiles, for patients most likely to benefit. The treatment algorithm described in this section reflects this personalised framework.

For patients with a CPS ≥10, pembrolizumab combined with chemotherapy (nab-paclitaxel, paclitaxel, or gemcitabine-carboplatin) represents the current first-line standard of care, based on the survival benefit demonstrated in KEYNOTE-355 (median OS 23.0 vs. 16.1 months; HR 0.73) [15,67]. In Europe, atezolizumab plus nab-paclitaxel is approved for first-line treatment of patients with PD-L1 positive tumours (IC ≥1%), following results from Impassion130, however, this indication was subsequently withdrawn by the FDA after confirmatory studies failed to demonstrate OS benefit [16]. The data from ASCENT-04/KEYNOTE-D19 presented at ASCO 2025 and demonstrating superiority of sacituzumab govitecan plus pembrolizumab over chemotherapy plus pembrolizumab in PD-L1-positive disease are anticipated to further reshape this first-line algorithm, potentially replacing chemotherapy as the backbone partner for immunotherapy [20].

For patients with a CPS <10 and IC <1% who lack actionable HRR alterations, the first-line standard remains single-agent or doublet chemotherapy, predominantly taxane-based or platinum-based regimens. However, results from ASCENT-03, and TROPION-Breast02 [19,22], may represent a paradigm-shifting option pending regulatory review.

For patients with germline *BRCA1/2* mutations, PARP inhibitors offer an alternative first-line or early-line option with a meaningful PFS benefit and a more favourable toxicity profile compared to chemotherapy. Activity has also been demonstrated in selected patients with germline *PALB2* mutations, and emerging data support consideration in somatic *BRCA1/2*-mutated disease, though the evidence base in these populations remains less mature and regulatory approvals vary by region. In the subset of patients who are both PD-L1-positive and *BRCA*-mutated, the optimal sequencing of PARP inhibitors versus ICIs remains unknown. Emerging data suggest that first-line ICI may be favoured, given evidence of diminished efficacy when ICIs are administered in later lines, whereas PARP inhibitors could be reserved for subsequent therapy in patients with actionable germline mutations. Such sequencing decisions are likely to be guided by tumour burden, patient comorbidities, and biomarker-driven risk stratification, with ongoing trials exploring combined or sequential PARP inhibitor plus ICI strategies.

Interpretation of the pivotal first-line ADC studies warrants specific methodological caution. Cross-trial comparisons between ASCENT-03 and TROPION-Breast02 are confounded by material differences in eligibility criteria. ASCENT-03 required a DFI of ≥6 months from curative treatment, selecting a relatively lower-risk relapsed population, whereas TROPION-Breast02 imposed no minimum DFI requirement and capped early relapsers (<12 months) at 20% of enrolment, thereby including a higher-risk cohort. TROPION-Breast02 additionally enrolled patients with stable brain metastases, expanding generalisability but further enriching for adverse prognostic features. Despite this, the comparator arm in TROPION-Breast02 only permitted single agent chemotherapy, whereas combination chemotherapy with gemcitabine and carboplatin was permitted and used in 44% on ASCENT-03. As a result, cross-trial PFS comparisons between these ADC strategies are unreliable in the absence of head-to-head data [19]. Crossover provisions further diverged: ASCENT-03 permitted crossover to SG at progression, with observed rates exceeding 80%, substantially confounding OS analysis and precluding definitive OS conclusions. In contrast TROPION-Breast02 did not permit crossover, enabling a cleaner OS readout but limiting post-progression treatment options for patients enrolled in regions without external access to datopotamab deruxtecan. The lower median PFS in the chemotherapy-pembrolizumab control arm of ASCENT-04 (7.8 months) compared with KEYNOTE-355 in the CPS ≥10 population (9.7 months) most plausibly reflects the higher proportion of patients with prior (neo)adjuvant chemotherapy exposure in ASCENT-04, including taxane-pretreated patients for whom the control regimen options may have had diminished efficacy, alongside differences in geographic enrolment and stratification variables, rather than true inferiority of the standard regimen [15,20]. These methodological heterogeneities underscore the need for standardised trial design elements in future first-line TNBC studies, including consistent DFI definitions, and pre-specified crossover-adjusted OS analyses, to enable robust cross-trial inference and support evidence-based treatment sequencing decisions.

CNS disease represents a major and incompletely addressed unmet need in metastatic TNBC. SG demonstrated minimal intracranial activity in the stable brain metastases subgroup of ASCENT (ORR 3%, median PFS 2.8 months), and the pharmacokinetic determinants of this limited CNS activity remain incompletely characterised [81]. The tetrapeptide-based cleavable linker of Dato-DXd confers greater plasma stability than SG’s hydrolysable CL2A linker [82], and ESG401’s serum-stable linker, designed to minimise premature payload release in circulation, may theoretically enhance CNS exposure compared with less stable constructs [85]. T-DXd has demonstrated substantial intracranial activity in HER2-positive breast cancer, most notably in the phase 3b/4 DESTINY-Breast12 trial (12-month CNS PFS 58.9%) [119], and HER3-DXd showed activity in leptomeningeal disease in TUXEDO-3, though the TNBC subgroup comprised only six patients [97]. Dedicated CNS-inclusive trials and mandatory reporting of intracranial efficacy endpoints in future first-line TNBC studies will be essential to address this critical gap.

Sequencing beyond the first line is increasingly complex and hindered by the absence of cross-trial comparisons and validated predictive biomarkers. SG remains the approved standard second-line therapy, demonstrating in ASCENT an ORR of 35%, median PFS of 5.6 months, and median OS of 12.1 months versus 5%, 1.7 months, and 6.7 months for TPC chemotherapy, respectively [17]. A major emerging challenge is the sequential use of two TROP-2-directed ADCs (SG and dato-DXd) given their shared topoisomerase I payload class, as cross-resistance mechanisms including MDR-associated efflux are likely to attenuate benefit from the second agent [153]. There is currently no phase III evidence supporting sequential TROP-2 ADC use, and active trials are seeking to address this gap.

A distinct sequencing consideration applies to patients with HER2-low or ultralow tumour expression. In this biomarker-defined subset, T-DXd offers an additional line of therapy in the pretreated setting based on DESTINY-Breast04 data [18], though its efficacy following prior SG or dato-DXd exposure remains unknown and prospective data addressing this sequence are awaited.

A further complexity arises from prior exposure to (neo)adjuvant immunotherapy. Patients who relapsed within six to twelve months of completing adjuvant pembrolizumab (following KEYNOTE-522) present a distinct clinical scenario in which re-challenging with checkpoint inhibition in the metastatic setting requires careful risk-benefit discussion, as primary resistance mechanisms may already be operative. This will be further complicated if ADCs are approved in the adjuvant setting in the future, although results from TROPION-Breast03 and ASCENT-05 are awaited. Treatment history review including prior ADC exposure, taxane class, and ICI timing is essential before selecting subsequent lines and participation in clinical trials should be encouraged.

**Figure 1:**
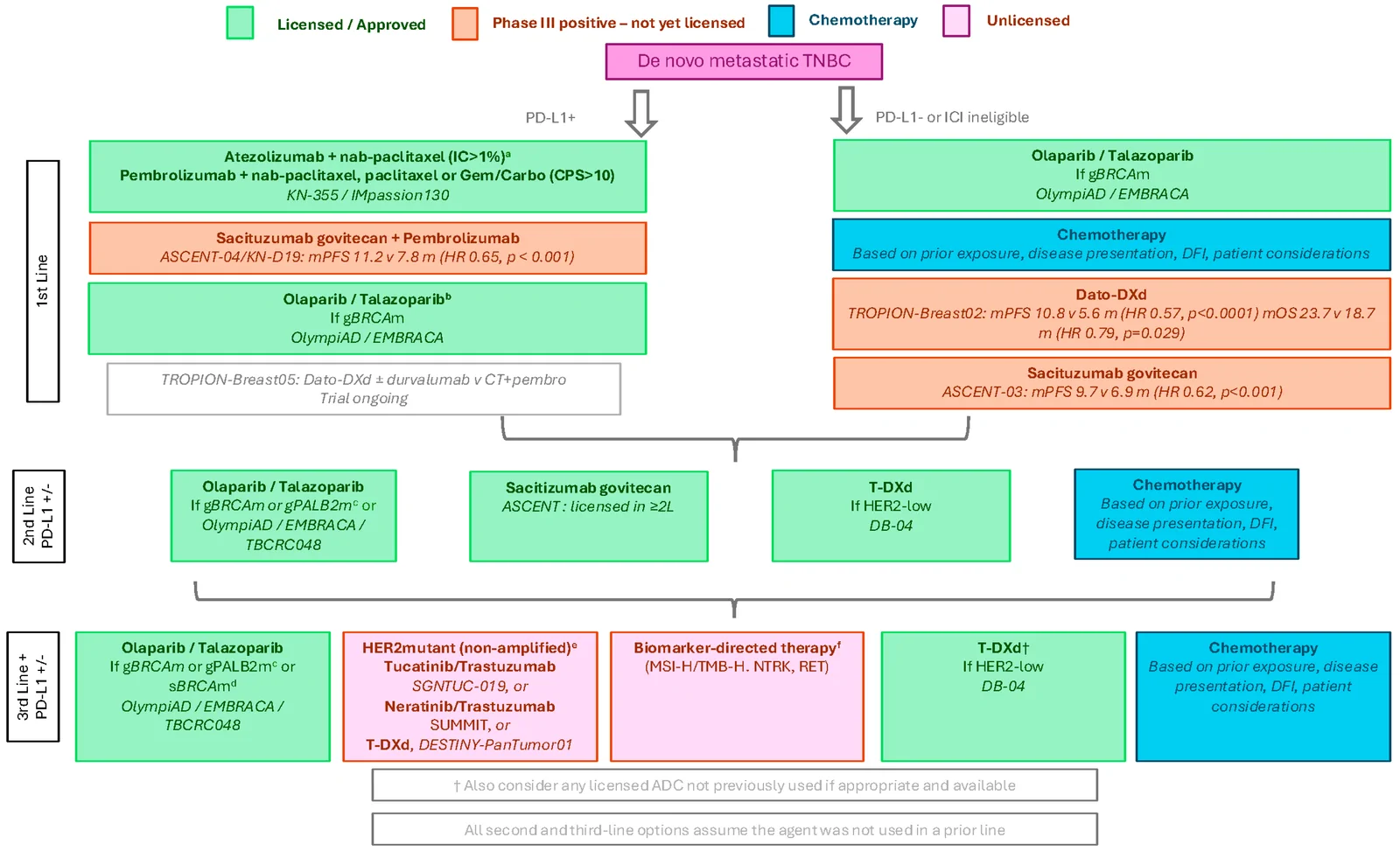
**Proposed treatment algorithm for *de novo* metastatic breast cancer.** ^a^Atezolizumab/nab-paclitaxel is EMA approved. FDA approval withdrawn 2021. ^b^In patients with both PD-L1-positive and g*BRCA1/2*-mutated disease, emerging data suggest that first-line ICI may be favoured, given evidence of diminished efficacy when ICIs are administered in later lines. PARP inhibitors may offer a reasonable alternative first-line option, particularly where a chemotherapy-free approach is preferred, and can be reserved for subsequent therapy. Optimal sequencing in this dual biomarker-positive population remains prospectively unevaluated and should be guided by individual patient factors including tumour burden, comorbidities, and treatment preferences. ^c^Germline *PALB2* pathogenic variant: olaparib NCCN Category 2A recommendation based on TBCRC-048 phase II data (ORR 75%, PFS 9.6 months); not FDA or EMA approved in this indication. Olaparib/talazoparib for g*BRCA1/2*-mutated disease: NCCN Category 1. ^d^s*BRCA1/2*: olaparib activity demonstrated in TBCRC-048 phase II trial; NCCN Category 2B recommendation; not FDA or EMA approved in this indication. ^e^Tucatinib + trastuzumab (SGNTUC-019) and neratinib + trastuzumab (SUMMIT) are both NCCN Category 2A recommendations for HER2-mutant non-amplified metastatic breast cancer; fulvestrant is omitted in ER-negative/TNBC patients. Neratinib without trastuzumab is Category 2B. Neither combination is FDA or EMA approved in this indication. T-DXd activity is supported by DESTINY-PanTumor01 (breast cancer subgroup ORR 50%); no current NCCN category or regulatory approval specifically for HER2-mutant non-amplified breast cancer. All options require HER2 activating mutation confirmed by NGS of tumour tissue or ctDNA; HER2 amplification should be excluded. NCCN Breast Cancer Guidelines version 5.2025. ^f^Tumour-agnostic FDA approvals exist for pembrolizumab (MSI-H and TMB ≥10 mut/Mb; KEYNOTE-158), larotrectinib/entrectinib (NTRK fusions), and selpercatinib (RET fusions); all carry NCCN Category 2A designation. These alterations are rare in TNBC (<1–5%) and breast cancer-specific efficacy data are limited. Comprehensive genomic profiling is recommended to identify eligible patients. None are EMA-approved or NICE-recommended specifically for TNBC. TNBC, triple-negative breast cancer; Dato-DXd, datopotamab deruxtecan (Datroway); DFI, disease-free interval; T-DXd, trastuzumab deruxtecan (Enhertu); CPS, combined positive score; IC, immune cell score; ICI, immune checkpoint inhibitor; mPFS, median progression-free survival; mOS, median overall survival; g*BRCA*, germline *BRCA*; g*PALB2*, germline *PALB2*; s*BRCA*, somatic *BRCA*; NGS, next-generation sequencing; ctDNA, circulating tumour DNA; MSI-H, microsatellite instability-high; TMB-H, tumour mutational burden-high; NTRK, neurotrophic tyrosine receptor kinase; RET, rearranged during transfection. Sequencing note: SG (TROP-2-directed, SN-38 payload) and Dato-DXd (TROP-2-directed, DXd payload) share a topoisomerase I inhibitor payload class; cross-resistance is a theoretical concern and sequential use data are limited. ***Approval status reflects regulatory submissions as of March 2026.***

**Figure 2:**
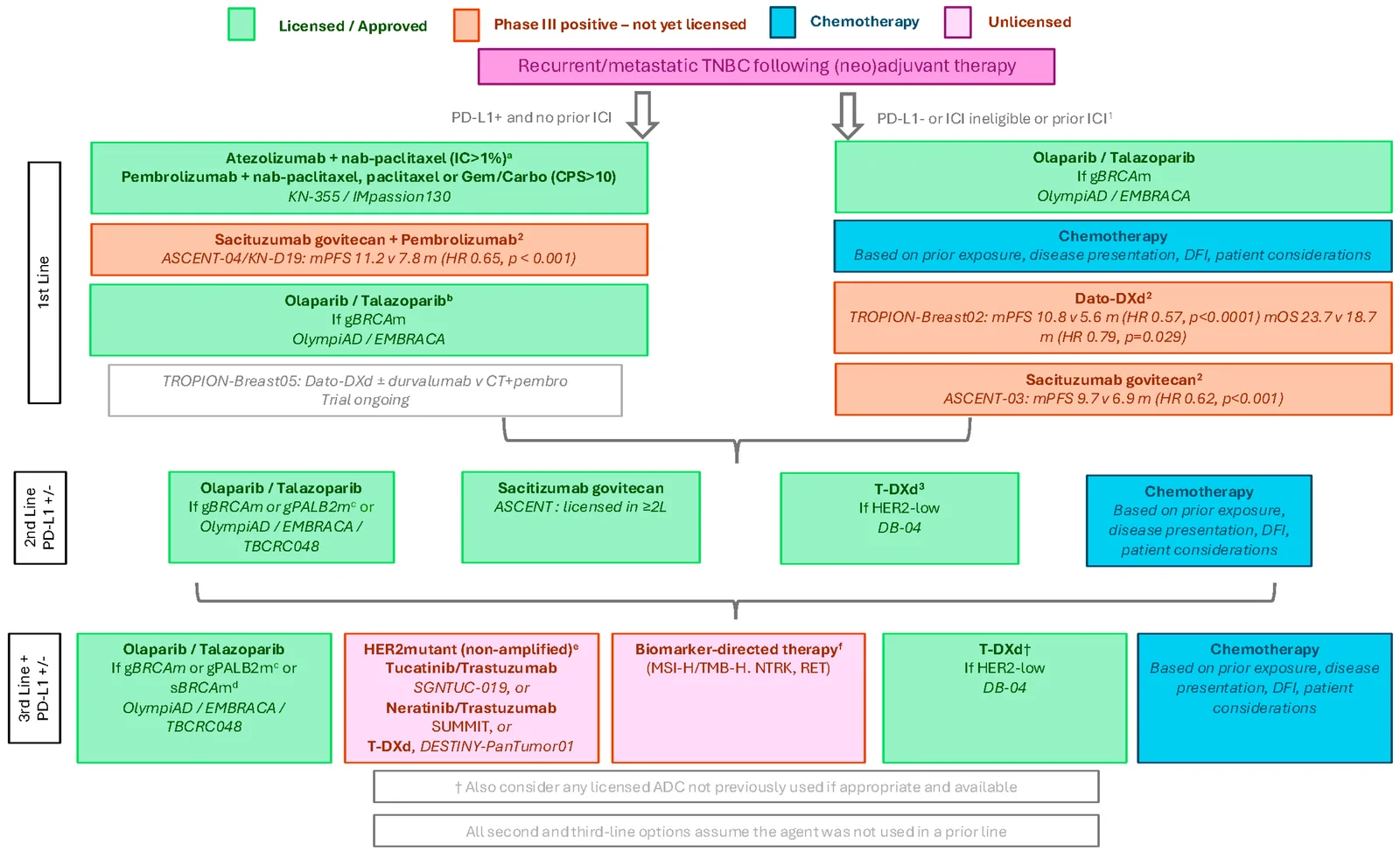
**Proposed treatment algorithm for metastatic breast cancer following (neo) adjuvant treatment.** ^1^For patients relapsing following adjuvant pembrolizumab (KEYNOTE-522), re-challenge with ICI in the metastatic setting remains clinically uncertain. ASCENT-04 permitted enrolment of patients with prior adjuvant ICI and stratified by this variable, however too few such patients were enrolled to draw conclusions regarding ICI re-challenge efficacy. The 6–12 month treatment-free interval is used as a pragmatic threshold in clinical practice; patients relapsing within 6 months of adjuvant ICI should be directed to the ICI-ineligible arm. For those relapsing beyond 12 months, individual patient discussion and clinical trial participation are encouraged given the absence of prospective evidence. ^2^Disease-free interval (DFI) requirements for recurrent disease: ASCENT-03 and ASCENT-04: DFI ≥6 months from completion of curative-intent therapy required. TROPION-Breast02: no minimum DFI requirement; patients with DFI <12 months capped at 20% of total enrolment. ^3^T-DXd may be used as first-line metastatic therapy in patients with HER2-low disease who relapse within 6 months of completing adjuvant chemotherapy (DESTINY-Breast04 eligibility criterion). ^a^Atezolizumab/nab-paclitaxel is EMA approved. FDA approval withdrawn 2021. ^b^In patients with both PD-L1-positive and g*BRCA1/2*-mutated disease, emerging data suggest that first-line ICI may be favoured, given evidence of diminished efficacy when ICIs are administered in later lines. PARP inhibitors may offer a reasonable alternative first-line option, particularly where a chemotherapy-free approach is preferred, and can be reserved for subsequent therapy. Optimal sequencing in this dual biomarker-positive population remains prospectively unevaluated and should be guided by individual patient factors including tumour burden, comorbidities, and treatment preferences. ^c^Germline *PALB2* pathogenic variant: olaparib NCCN Category 2A recommendation based on TBCRC-048 phase II data (ORR 75%, PFS 9.6 months); not FDA or EMA approved in this indication. Olaparib/talazoparib for g*BRCA1/2*-mutated disease: NCCN Category 1. ^d^s*BRCA1/2*: olaparib activity demonstrated in TBCRC-048 phase II trial; NCCN Category 2B recommendation; not FDA or EMA approved in this indication. ^e^Tucatinib + trastuzumab (SGNTUC-019) and neratinib + trastuzumab (SUMMIT) are both NCCN Category 2A recommendations for HER2-mutant non-amplified metastatic breast cancer; fulvestrant is omitted in ER-negative/TNBC patients. Neratinib without trastuzumab is Category 2B. Neither combination is FDA or EMA approved in this indication. T-DXd activity is supported by DESTINY-PanTumor01 (breast cancer subgroup ORR 50%); no current NCCN category or regulatory approval specifically for HER2-mutant non-amplified breast cancer. All options require HER2 activating mutation confirmed by NGS of tumour tissue or ctDNA; HER2 amplification should be excluded. NCCN Breast Cancer Guidelines version 5.2025. ^f^Tumour-agnostic FDA approvals exist for pembrolizumab (MSI-H and TMB ≥10 mut/Mb; KEYNOTE-158), larotrectinib/entrectinib (NTRK fusions), and selpercatinib (RET fusions); all carry NCCN Category 2A designation. These alterations are rare in TNBC (<1–5%) and breast cancer-specific efficacy data are limited. Comprehensive genomic profiling is recommended to identify eligible patients. None are EMA-approved or NICE-recommended specifically for TNBC. TNBC, triple-negative breast cancer; Dato-DXd, datopotamab deruxtecan (Datroway); DFI, disease-free interval; T-DXd, trastuzumab deruxtecan (Enhertu); CPS, combined positive score; IC, immune cell score; ICI, immune checkpoint inhibitor; mPFS, median progression-free survival; mOS, median overall survival; gBRCA, germline *BRCA*; g*PALB2*, germline *PALB2*; s*BRCA*, somatic *BRCA*; NGS, next-generation sequencing; ctDNA, circulating tumour DNA; MSI-H, microsatellite instability-high; TMB-H, tumour mutational burden-high; NTRK, neurotrophic tyrosine receptor kinase; RET, rearranged during transfection. Sequencing note: SG (TROP-2-directed, SN-38 payload) and Dato-DXd (TROP-2-directed, DXd payload) share a topoisomerase I inhibitor payload class; cross-resistance is a theoretical concern and sequential use data are limited. ***Approval status reflects regulatory submissions as of March 2026.***

See Fig. 1 and Fig. 2 for proposed treatment algorithms for metastatic TNBC.

### 10.2 Toxicity Management and Quality of Life

As the treatment landscape evolves, managing overlapping and cumulative toxicities across sequential agents has become an increasingly important clinical priority. Immunotherapy-related adverse events, including immune-mediated thyroid dysfunction, pneumonitis, colitis, hepatitis, and adrenal insufficiency, occur in approximately 25% of patients receiving chemoimmunotherapy, with grade 3–4 events in around 5% [15]. Prompt recognition, early corticosteroid administration, and multidisciplinary input are critical. An additional consideration in the era of ADC-ICI combinations is the potential for overlapping pulmonary toxicity. ILD is a class effect of DXd-based ADCs and has been reported in up to approximately 5% of patients treated with dato-DXd in the phase III setting, requiring vigilant monitoring and dose interruption protocols [103].

Haematological toxicity, particularly neutropenia and anaemia, is universal across cytotoxic regimens. SG is associated with clinically significant neutropenia (grade 3–4 in approximately 51% of patients) and diarrhoea (grade 3–4 in approximately 10%), which may be managed with G-CSF support and loperamide, respectively [17]. PARP inhibitors are associated with anaemia and thrombocytopenia, which are generally manageable but require regular monitoring, particularly in patients with reduced bone marrow reserve following prior platinum-based therapy. Long-term peripheral neuropathy from taxane and eribulin exposure may limit patients’ capacity for subsequent neurotoxic agents and must be routinely assessed.

Patient-reported outcomes (PROs) and quality of life (QoL) data are increasingly incorporated into regulatory submissions and trial reporting. IMpassion130 demonstrated that the addition of atezolizumab to nab-paclitaxel did not compromise health-related QoL [16]. Similarly, ASCENT-04 included QoL endpoints, with preliminary data suggesting that SG plus pembrolizumab maintained QoL compared to chemotherapy-immunotherapy in PD-L1-positive disease [20]. These data are particularly relevant given the chronic nature of metastatic disease management, in which prolonged toxicity may offset survival gains. 

A patient-centred approach incorporating individual comorbidities, functional status, treatment preferences, and logistical considerations, including the convenience of oral versus intravenous administration, should inform treatment selection at every line.

### 10.3 Resistance Mechanisms

Resistance to treatment remains the central challenge in the management of metastatic TNBC, and its mechanisms are as heterogeneous as the disease itself. Resistance to chemotherapy arises through multiple mechanisms, including upregulation of drug efflux pumps (P-glycoprotein/MDR1), alterations in DNA repair pathways, EMT conferring stem-cell-like properties, and dysregulation of the PI3K/AKT/mTOR pathway that promotes cell survival under cytotoxic stress [154]. The dense stromal microenvironment further limits drug penetration and facilitates immune exclusion.

Resistance to ICIs in TNBC is mediated through both intrinsic tumour-cell mechanisms and extrinsic microenvironmental factors. Intrinsic mechanisms include loss of MHC class I expression, dysregulation of interferon-gamma signalling, oncogenic activation of Wnt/beta-catenin and JAK/STAT pathways, and epigenetic silencing of immunogenic gene expression programmes [154,155]. Critically, these mechanisms interact with the genomic landscape of TNBC. *TP53* loss, which is present in up to 80% of cases, promotes chromosomal instability that can paradoxically exhaust cGAS-STING-mediated innate immune sensing, attenuating the interferon signalling that underpins ICI responsiveness [156,157]. TGF-β pathway activation, frequently upregulated within the immunosuppressive stroma of TNBC, independently suppresses cytotoxic T-lymphocyte function and drives acquired ICI resistance through a mechanism not addressed by PD-1/PD-L1 blockade alone [49], providing a mechanistic rationale for ongoing trials of TGF-β/PD-L1 bifunctional agents in this disease. Extrinsically, expansion of immunosuppressive populations including regulatory T cells (Tregs), myeloid-derived suppressor cells (MDSCs), and M2-polarised tumour-associated macrophages (TAMs), and secretion of immunosuppressive cytokines such as TGF-beta and IL-10 collectively attenuate effector T-cell function [158].

ADC resistance mechanisms differ importantly by drug class and cannot be considered a uniform phenomenon. A pivotal preclinical study by Rampa et al. [159] demonstrated that resistance to both T-DXd and SG was driven primarily by upregulation of payload-specific efflux transporters rather than loss of target antigen expression, and that switching to ADCs with mechanistically distinct payloads, specifically microtubule inhibitors, restored antitumour activity in resistant models *in vitro* and *in vivo* [159]. These findings have direct sequencing implications. Because SG and Dato-DXd share a topoisomerase I inhibitor payload class (SN-38 and DXd respectively), cross-resistance mediated by shared efflux pathway upregulation is mechanistically plausible when one is used sequentially after the other, and retrospective data demonstrate substantially diminished efficacy when sequential TROP-2-directed ADCs are used, with markedly shorter time-to-treatment-failure for the second ADC compared with the first irrespective of order [160]. By contrast, the resistance profile of T-DXd is mechanistically distinct, characterised by HER2 antigen downregulation or binding-site mutation at the protein level, and by ABCC1 (MRP1)-mediated DXd efflux, operating independently of TROP-2 antigen status [161,162]. This mechanistic divergence explains why sequential use of a TROP-2-directed ADC after T-DXd, switching both the antibody target and exploiting residual DXd payload sensitivity, represents a more biologically rational strategy than sequential use of two Topo I-based TROP-2 ADCs, despite the shared payload class of the latter pair [159,162]. Antigen downregulation and impaired antibody internalisation remain relevant resistance mechanisms for TROP-2-directed agents, and the bystander killing effect of cleavable-linker ADCs partially compensates for antigen heterogeneity without fully overcoming efflux-mediated resistance [153].

PARP inhibitor resistance in *BRCA*-mutated TNBC arises through several mechanistically distinct pathways. Secondary *BRCA1/2* reversion mutations, which are somatic insertions or deletions that restore the open reading frame and reconstitute functional HR protein, represent the dominant mechanism, detected in up to 60% of patients with acquired resistance by longitudinal ctDNA profiling [163]. Reversion mutations confer simultaneous cross-resistance to platinum and PARPi, reflecting their shared dependence on HR deficiency. ctDNA detection of a reversion prior to subsequent therapy has been associated with significantly shorter time to progression, supporting a role for liquid biopsy monitoring at disease progression [163,164]. Non-reversion mechanisms, including loss-of-function alterations in *TP53BP1*, *RIF1*, and *PAXIP1*, secondary mutations in *RAD51C*, *RAD51D*, and *PALB2*, and epigenetic resistance via loss of BRCA1 promoter hypermethylation, which reactivates silenced BRCA1 transcription, account for a substantial minority of cases and can co-occur with reversion mutations [164,165]. Downregulation of PARP1 expression itself represents a further target-level resistance mechanism independent of HR restoration [163,165,166].

### 10.4 Biomarker-Driven Treatment Selection: Opportunities and Limitations

Despite the proliferation of new agents, the majority of patients with metastatic TNBC, particularly those who are PD-L1-negative and lack HRR alterations, remain without a validated biomarker-driven treatment strategy beyond broad-population ADCs. While PARP inhibitors are approved for germline *BRCA1/2*-mutated disease and may have activity in selected patients with somatic *BRCA* or germline *PALB2* mutations, these populations represent a minority of TNBC cases. PDL1 testing is complicated by inter-assay variability, and the CPS threshold of ≥10 for pembrolizumab excludes approximately 60% of patients.

The implementation of biomarker-driven therapy selection faces important technical challenges. PD-L1 testing requires non-interchangeable assay-antibody-scoring pairings. Atezolizumab requires SP142 with IC ≥1%, whilst pembrolizumab requires 22C3 with CPS ≥10, with only approximately 75–80% concordance between the two assays [167], meaning treatment eligibility may differ depending solely on which assay is performed. When results are indeterminate or technically inadequate, repeat biopsy or reflex testing with the alternative assay should be considered before classifying a patient as ICI-ineligible. HER2-low designation introduces analogous challenges, with substantial inter-observer variability at the IHC 1+ threshold. Analysis from DESTINY-Breast04 suggest that central pathology review may reclassify a proportion of tumours initially scored as HER2-0 in local practice as HER2-low, indicating potential underestimation of eligibility in routine settings [168]. Where T-DXd is being considered on the basis of a community IHC 0 result, repeat or central HER2 assessment is warranted. Use of FDA-approved companion diagnostics in preference to laboratory-developed tests remains the standard for clinical decision-making where available.

Circulating tumour DNA (ctDNA) has emerged as a promising non-invasive biomarker for real-time monitoring of tumour dynamics, early detection of molecular resistance, and prognostication in metastatic cancer, including TNBC. ctDNA reflects tumour burden, and changes in ctDNA levels often precede radiographic or clinical evidence of progression, offering an opportunity for earlier therapeutic decision-making. Low baseline ctDNA levels and early suppression or clearance of ctDNA during therapy have been associated with improved PFS and ORR across multiple targeted and systemic therapies [169].

Spatial transcriptomic and single-cell sequencing enable high-resolution mapping of tumour cellular heterogeneity and immune microenvironment organisation while preserving spatial context. These technologies provide insights into immune evasion mechanisms, stromal–immune interactions, and determinants of response and resistance in TNBC, although their current application remains primarily exploratory [170].

The FUTURE-SUPER trial provided proof-of-concept that biomarker-guided, molecularly stratified therapy may improve outcomes compared with standard chemotherapy across TNBC subtypes. This phase II umbrella study used genomic and transcriptomic profiling to assign patients to subtype-directed regimens, demonstrating an improvement in PFS compared with non-stratified chemotherapy. However, the study was exploratory and underpowered for definitive conclusions, and did not incorporate immune checkpoint blockade in the standard comparator pathway for PD-L1–positive disease [171]. These findings nevertheless support the potential value of umbrella trial designs in refining precision treatment strategies in TNBC.

### 10.5 Access, Equity, and Health Economic Considerations

The introduction of novel ADCs, ICI combinations, and targeted agents has substantially increased the cost of treating metastatic TNBC. Pembrolizumab, SG, T-DXd, PARP inhibitors and the emerging combination regimens all carry significant per-cycle costs that limit accessibility in lower-resource settings and present challenges for national health technology assessment bodies. Emerging strategies to improve the affordability of ICIs in resource-constrained settings are currently being explored. The phase II PLANeT trial presented at ESMO 2025 evaluated a reduced dose pembrolizumab regimen in combination with standard NACT in early TNBC. This study suggested that lower fixed doses of pembrolizumab (50 mg three-weekly for 3 cycles) may achieve pharmacokinetic exposure and preliminary clinical activity comparable to the standard 200-mg schedule. pCR was higher with low-dose pembrolizumab plus NACT vs. NACT alone group (53.8% v 40.5%, *p* = 0.047) [172]. This magnitude of benefit is numerically similar to historical data from standard-dose pembrolizumab in KN-522. Although these findings require validation in larger randomised trials, dose-optimisation strategies could substantially reduce treatment costs and expand access to ICIs in resource-limited healthcare systems. If confirmed, such approaches may represent an important step toward improving global equity in the management of metastatic TNBC.

The populations who bear the greatest burden of metastatic TNBC, particularly Black women and other racial/ethnic minorities, remain systematically underrepresented in the pivotal trials that define current standards of care. In ASCENT, KEYNOTE-355, and DESTINY-Breast04, Black patients comprised fewer than 10% of participants despite representing a disproportionate share of the TNBC population, and dedicated racial subgroup efficacy analyses have not been reported for most pivotal trials. Available real-world data suggest that Black women with metastatic TNBC derive clinical benefit from SG comparable to the overall ASCENT population [173]. Strategies to improve trial representation, including community-based recruitment, removal of overly restrictive eligibility criteria, and decentralised trial participation, are necessary to ensure that the evidence base reflects the populations most affected by this disease.

Ensuring equitable access to biomarker testing, including germline *BRCA* testing, PD-L1 IHC, and HER2 low re-assessment, and to novel therapies must be a parallel priority alongside clinical development. Risk-sharing agreements, biosimilar development, and adaptive healthcare funding models will be critical to translating the scientific advances in metastatic TNBC into population-level benefit.

### 10.6 Adaptive Trial Designs and Regulatory Innovation

The growing number of emerging agents and combination strategies in metastatic TNBC cannot be evaluated efficiently through conventional sequential phase III trials. Adaptive platform and umbrella trials such as the FUTURE series and SUMMIT basket trial enable simultaneous assessment of multiple therapies in molecularly defined subtypes, often using response-adaptive randomisation and seamless phase II/III designs. Master protocols incorporating ctDNA-defined cohorts provide additional efficiencies. Regulatory agencies, including the FDA and EMA, increasingly support accelerated approvals based on surrogate endpoints (ORR, PFS) in pretreated populations, with confirmatory OS data required post-approval.

Basket trials targeting shared oncogenic alterations further expand treatment options for molecularly selected TNBC patients, independent of histology. Decentralised and hybrid trial designs, incorporating remote participation and digital biomarker monitoring, may accelerate enrolment and improve access for underrepresented populations. Embedding translational sub-studies, including mandatory biopsies at baseline and progression, is critical to elucidate resistance mechanisms and identify predictive biomarkers. International collaborative networks and data-sharing frameworks will be essential to achieve the sample sizes and molecular resolution required to address the complex questions facing the field.

## 11 Conclusions

The treatment landscape of metastatic TNBC has undergone a remarkable and accelerating transformation over the past decade. From an era defined by sequential monotherapy chemotherapy as the sole systemic option, yielding a median OS of twelve to eighteen months, the field has entered a new paradigm driven by the convergence of ICI, ADCs, and molecularly targeted agents. Three distinct drug classes have now demonstrated PFS benefit in randomised trials: PARP inhibitors in germline *BRCA*-mutated disease (and in selected patients with somatic *BRCA* or germline *PALB2* mutations), ICI in combination with chemotherapy in PD-L1-positive disease, and SG across molecularly unselected pretreated populations, with TDXd extending options to the HER2-low subpopulation. The positive results of ASCENT-04/KEYNOTE-D19, ASCENT-03, and TROPION-Breast02 presented in 2025, signify a further paradigm shift that may establish ADC-based first-line therapy, with or without checkpoint inhibition, as the new standard of care, potentially displacing conventional chemotherapy as the anchor for first-line treatment.

Yet the translation of these advances into durable, broadly accessible benefit for all patients with metastatic TNBC remains an urgent and incompletely realised goal. The majority of patients who are PD-L1-negative and *BRCA* wild-type still lack validated biomarker-driven options beyond chemotherapy. Resistance, whether primary or acquired, to ICI, ADCs, and targeted agents inevitably limits the durability of response, and the optimal sequencing of multiple active classes across treatment lines remains empirically unresolved. The development of shared topoisomerase I class payloads across multiple ADCs raises questions of cross-resistance that prospective clinical trials must address. Structural inequities in biomarker testing access and therapeutic availability continue to limit the real-world impact of clinical advances, particularly for populations with the highest TNBC incidence and the least access to healthcare resources.

Looking forwards, the integration of molecular subtyping, liquid biopsy-guided dynamic monitoring, and rational combination strategies offer the most credible path toward improving long-term outcomes. Adaptive platform trial designs capable of rapidly evaluating emerging combinations in biomarker-defined populations will be essential to keep pace with the remarkable breadth of agents in late-stage development, including novel payload ADCs, bispecific antibodies, cancer vaccines, PARP1-selective inhibitors, and cell-based therapies. Continued investment in translational research, equitable access to genomic diagnostics, and meaningful inclusion of under-represented populations in clinical trials are not only scientific priorities but moral imperatives in a disease that disproportionately affects young women in the prime of life.

In summary, metastatic TNBC has evolved from a uniformly chemotherapy-treated disease to a biomarker-stratified therapeutic landscape shaped by ICIs, PARP inhibitors, and ADCs. While these advances have improved outcomes for defined subgroups, substantial unmet need persists, particularly among patients without actionable biomarkers and those with treatment-refractory or CNS disease. Resistance to current therapeutic classes and uncertainty around optimal sequencing continue to limit the durability of benefit across the disease course. Future progress will depend on refined biomarker development, rational combination and sequencing strategies, and resistance-informed therapeutic development supported by robust translational science and global collaborative clinical trials.

## Data Availability

Not applicable.
